# REST Controls Self-Renewal and Tumorigenic Competence of Human Glioblastoma Cells

**DOI:** 10.1371/journal.pone.0038486

**Published:** 2012-06-11

**Authors:** Luciano Conti, Laura Crisafulli, Valentina Caldera, Monica Tortoreto, Elisa Brilli, Paola Conforti, Franco Zunino, Lorenzo Magrassi, Davide Schiffer, Elena Cattaneo

**Affiliations:** 1 Department of Pharmacological Sciences and Centre for Stem Cell Research, Università degli Studi di Milano, Milano, Italy; 2 Neuro-bio-oncology Center of Policlinico di Monza Foundation, Vercelli, Italy; 3 Fondazione IRCCS Istituto Nazionale dei Tumori, Milan, Italy; 4 Neurochirurgia, Dipartimento di Scienze Chirurgiche, Università di Pavia Fondazione IRCCS Policlinico S. Matteo, Pavia, Italy; Sanford-Burnham Medical Research Institute, United States of America

## Abstract

The Repressor Element 1 Silencing Transcription factor (REST/NRSF) is a master repressor of neuronal programs in non-neuronal lineages shown to function as a central regulator of developmental programs and stem cell physiology. Aberrant REST function has been associated with a number of pathological conditions. In cancer biology, REST has been shown to play a tumor suppressor activity in epithelial cancers but an oncogenic role in brain childhood malignancies such as neuroblastoma and medulloblastoma. Here we examined REST expression in human glioblastoma multiforme (GBM) specimens and its role in GBM cells carrying self-renewal and tumorigenic competence. We found REST to be expressed in GBM specimens, its presence being particularly enriched in tumor cells in the perivascular compartment. Significantly, REST is highly expressed in self-renewing tumorigenic-competent GBM cells and its knock down strongly reduces their self-renewal *in vitro* and tumor-initiating capacity *in vivo* and affects levels of miR-124 and its downstream targets. These results indicate that REST contributes to GBM maintenance by affecting its self-renewing and tumorigenic cellular component and that, hence, a better understanding of these circuitries in these cells might lead to new exploitable therapeutic targets.

## Introduction

Glioblastoma multiforme (GBM) represents the most frequent and malignant cerebral neoplasia. Recent research has reported the presence of a population of cells defined as Glioblastoma Stem Cells (GSCs) because of their tumorigenic potential and similarities to the Neural Stem cells (NSCs) (i.e. *in vitro* growth conditions, self-renewal, expression of stem cell markers and differentiative, although aberrant, multipotency) [1], [2], [3]. These cellular elements have been suggested to display high chemo- and radio-resistance features, so that cells escaping surgical removal can regenerate the tumor despite adjuvant therapies. The elucidation of the molecular circuitries that control their self-renewal and tumorigenic competence can therefore likely foster the development of new targeted therapeutic approaches.

The RE1-silencing transcription factor (REST) [also known as neuron-restrictive silencer factor (NRSF)] has been characterized as a master repressor of neuronal programmes in non-neuronal tissues [4], [5], [6], [7]. REST binds to a highly conserved 21–23 bp DNA sequence called RE1 (repressor element 1) located in many neuronal genes and silences their transcription by recruiting specific co-repressors multicomplexes [8]. REST targets include a large number of genes that encode for neuron-specific proteins. Genome- and transcriptome-wide analyses focused on the RE-1 sequence have revealed a REST-regulated network with over 1000 genes and several microRNAs in humans [9], [10], [11]. REST has been shown to coordinate neural induction and neuronal differentiation programs during both *in vivo* and *in vitro* neurodevelopment [12], [13], [14], [15]. Of note interfering with REST levels leads to altered NSCs differentiation and aberrant REST function has been associated with neurological disorders [16], [17], [18], [19]. In human epithelial cells REST has been described as a potent suppressor of malignant transformation [20] and its deregulation has been associated with several non-neural tumors including breast and small cell lung cancers [21], [22]. However, oncogenic roles for REST have been observed in neuroblastoma [23] and medulloblastoma [24]. To date, although a certain level of REST amplification was reported in 36% of GBM specimens [25] and a study indicated expression of REST in GBM cells [26], whether REST levels and functions play critical roles in GBM yet represents a critical issue. Here, we report that REST is expressed in human GBM specimens and that its downregulation strongly impairs self-renewal and tumor-initiating capacity of GBM cells. These results indicate that REST plays critical functions in human GBM.

## Results and Discussion

### REST Expression is Elevated in Human GBM

**Figure 1:**
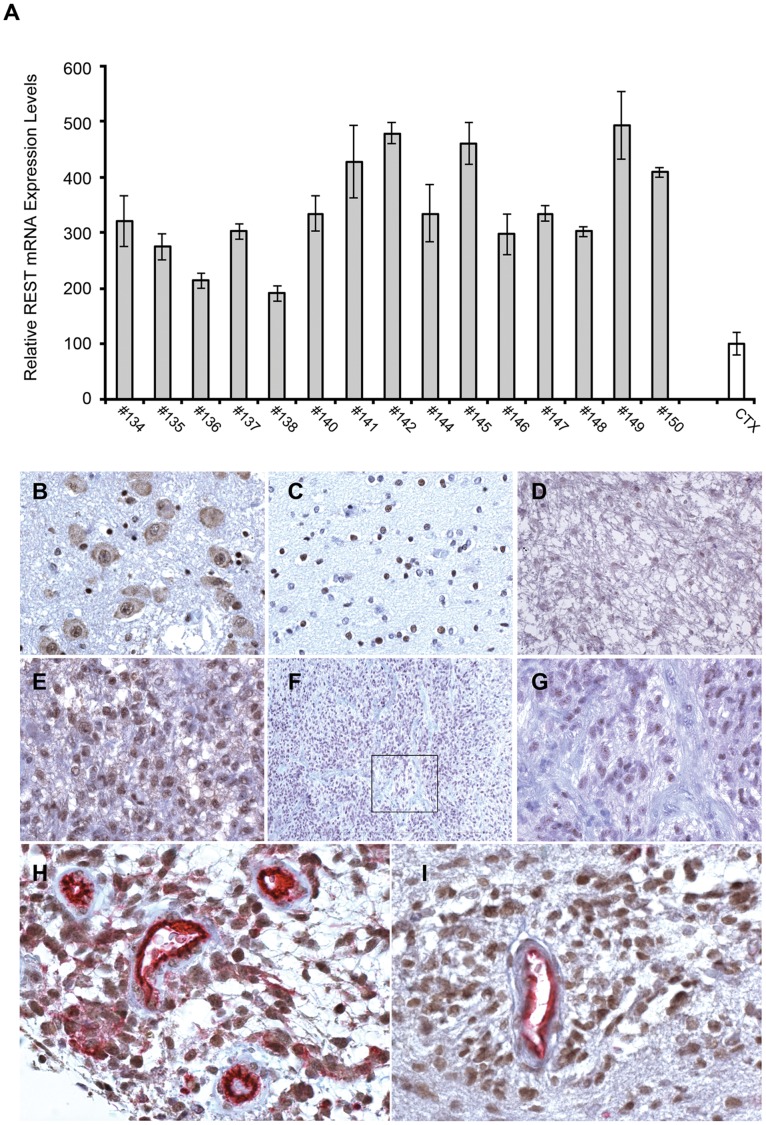
REST expression is elevated in human GBM. (A) Quantitative RT-PCR analysis of REST mRNA level, relative to GAPDH, in human primary GBM specimens compared to pooled non pathologic human cerebral cortex tissues (CTX). Results are relative to three independent experiments. Data are means ± s.d. and were analyzed with Student’s t-test. (B-I) Representative images of immunohistochemical staining of REST in human non pathologic brain tissue and glioma specimens. (B) Neurons of pontine nuclei with nuclear and cytoplasmic staining. DAB, 400×. (C) Glial nuclei in the hemispheric white matter. DAB, 400×. (D) Diffuse astrocitoma: few nuclei are weakly positive. DAB, 200×. (E) GBM: all the nuclei in peculiar proliferating areas are intensely positive. DAB, 400×. (F) GBM: nuclei of hypercellular areas with high vessel density are intensely positive. DAB, 100×. (G) Inset of (f). DAB, 400×. (H and I) GBM: intense nuclear staining in perivascular cell cuffings for REST and Sox2 respectively (vascular tissue is revealed by CD-31 immunoreactivity). Double staining (REST-CD31, Sox2-CD31), 400×.

To investigate if REST plays a functional role in GBM, we first evaluated, by quantitative real-time PCR assay, REST expression levels in 15 tumor specimens collected from independent primary GBMs patients. In all samples, REST is expressed and upregulated from two- to five-fold as compared to control mRNA obtained from pooled adult normal cerebral cortex tissues (Figure 1A). Interrogation of the National Cancer Institute’s Repository for Molecular Brain Neoplasia Data (REMBRANDT) [27] also indicated a 45% average increase of REST expression in gliomas compared to control (non-tumor) tissue (Figure S1A). As elevated levels of mRNA may reflect an increase in gene copy number, we sought to determine whether genomic changes in *REST* occurred in glioma patients. Copy number REMBRANDT analysis for *REST* indicated a 3-fold or greater amplification of the 4q12 chromosomal region which correlated with a slightly worse survival, while a deletion is strongly associated to a better survival (Figure S1B). To confirm the high REST expression in GBM tissues and to extend the analysis to protein level and REST cellular distribution, we performed an immunohistochemistry screening on 96 paraffin-embedded primary human brain tumor surgical biopsy specimens (Table 1). A detailed mapping of REST positive cells in diverse brain tumors has not been reported in the literature. In the control specimens, REST immunoreactivity was detected in the cytoplasm of selected neurons in the cerebral cortex and in dentate gyrus of hippocampus (Figure 1B). Glial cells diffusely showed a nuclear staining (Figure 1C). In neuroepithelial and non-neuronal tumors, REST immunoreactivity variably marked different cell populations, although with variable degrees both in terms of intensity and percentage of positive cells (Table 1; Figure S2A-F). In general, gliomas exhibited a nuclear staining which intensity increased with anaplasia (Figure 1D and E; Figure S2). REST was detected in all GBM specimens (the number of positive tumor cells differed between individual cases, ranging from 10 to 75%; Table 1) in phenotypic areas characterized by high cell density, small and hyperchromatic nuclei (Figure 1F and G). Immunoreactivity appeared as a strong nuclear-restricted staining, particularly intense in the perivascular districts (Figure 1H), areas known to be characterized by the highest malignancy and which also show high expression of Sox2 (Figure 1I) and nestin (not shown). Interrogation of the Human Protein ATLAS database (www.proteinatlas.org/) confirmed the same REST immunoreactive signal and cellular distribution, obtained with different antibodies with respect to the one here employed.

**Table 1:** Intensity and distribution of REST expression and demographic data (male:female ratio, mean age, age range) of the tumor specimens analysed in this study.

Tumor diagnosis	Total cases	Male: female ratio	Mean age at diagnosis (years)	Age range(years)	REST^+ve^Cells (%)	Intensity
**Glioblastoma Multiforme**	60	37∶23	62	23–81	10–75%	++/+++
**Diffuse astrocytoma**	3	2∶1	47	38–56	10–20%	+
**Anaplastic astrocytoma**	6	4∶6	66	51–75	30–50%	+
**Pilocytic astrocytoma**	6	2∶4	45	19–65	100%	++
**Oligodendroglioma**	10	5∶5	55	45–67	10–20%	+
**Anaplastic oligodendroglioma**	7	4∶3	55	31–80	30–40%	+/++
**Medulloblastoma**	3	2∶1	35	32–39	10–50%	+/++
**Central neurocytoma**	1	Female	22	/	positive	+
**Normal nervous tissue**	2	1∶1	46	43–49	/	/

### Elevated REST Expression is Maintained in Human Tumorigenic-competent GBM Cells

**Figure 2:**
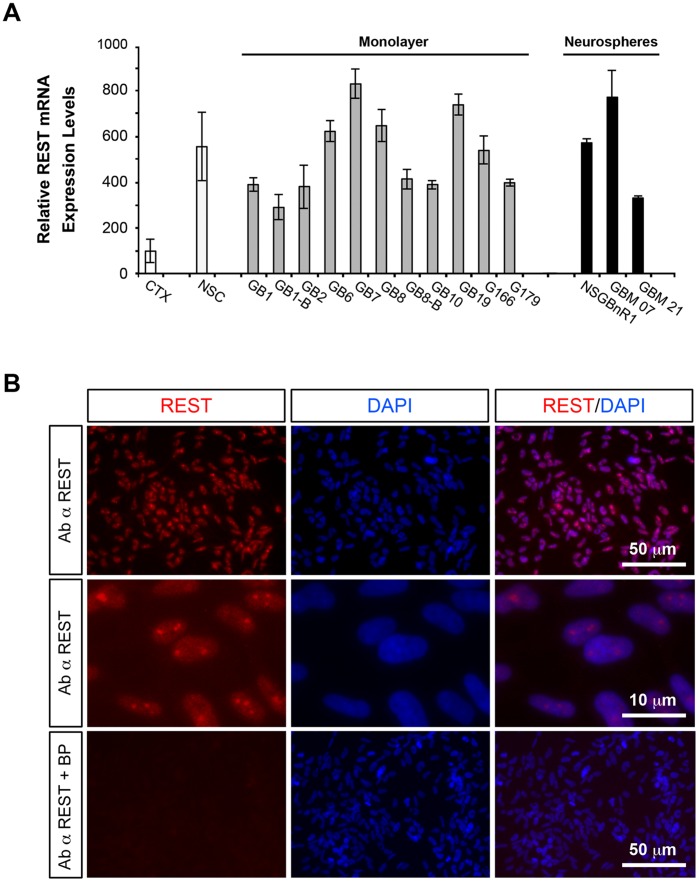
Elevated REST expression is maintained in human tumorigenic-competent GBM cells. (A) Quantitative RT-PCR analysis of REST mRNA level, relative to GAPDH, in different human GB lines grown as monolayer cultures or floating neurospheres compared to pooled non pathologic human cerebral cortex tissues (CTX) and to normal fetal-derived human Neural Stem Cells (NSCs). Data were analyzed with Student’s *t*- test. Results are relative to three independent experiments. Data are means ± s.d. (B) REST immunofluorescent staining of a representative GB cell line (GB7 cells). All the cells show diffuse REST nuclear signal. Signal specificity is demonstrated by competition with a specific REST blocking peptide (BP; bottom line).

Since previous studies suggested that the perivascular region is enriched for cellular elements defined as Glioblastoma Stem Cells (GSCs) because of their tumorigenic potential and some similarities to the Neural Stem cells (NSCs) [28], we assayed REST expression levels in self-renewing and tumorigenic competent cell populations that we have established from primary GBMs. Quantitative real-time PCR assay confirmed high level of REST mRNA expression, comparable to those observed in normal human fetal brain-derived NSCs and consistently higher compared to adult normal (non-pathological) brain tissue, in all the analyzed GBM-derived cells cultured in mitogen-supplemented Serum Free (SFM) conditions typically employed for growing NSCs (Figure 2A), both as adherent monolayer [29] (here named “GB cells”; for a characterization of GB cells see Figures S3 and S4) or as neurospheres. Immunocytochemistry confirmed the presence of REST nuclear signal in GB cells (Figure 2B). Noteworthy, a strong dotted staining could be observed in the nuclei, likely corresponding to nuclear foci, where co-expression of REST and telomere repeat factor 2 (TRF-2) has been previously reported [26].

### REST Knockdown Reduces Self-renewal Potential of Human Tumorigenic-competent GBM Cells and Induces Neuronal Differentiation and Cell Death Programs

**Figure 3:**
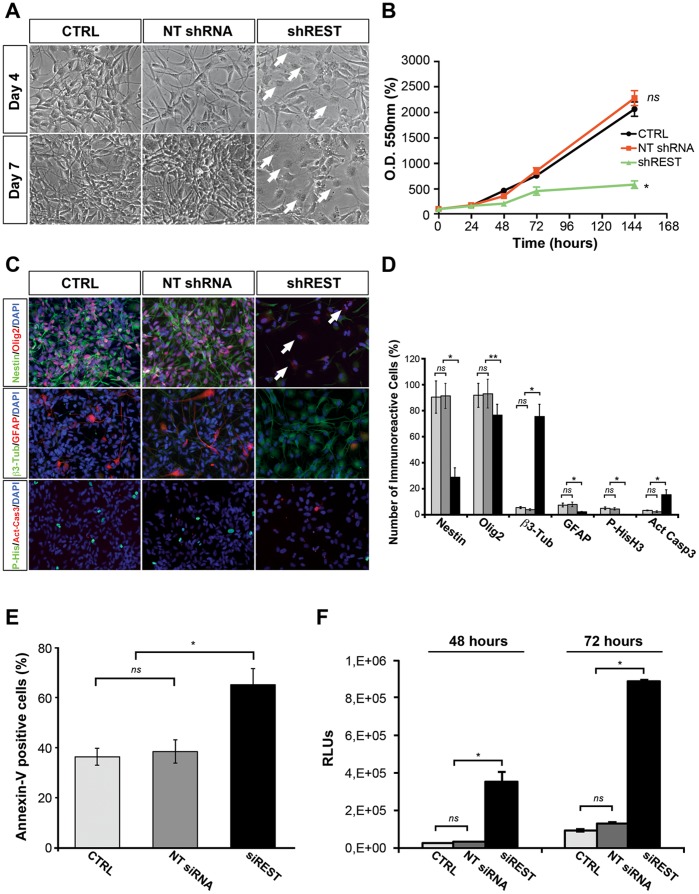
REST knockdown reduces self-renewal potential of human tumorigenic-competent GBM cells and induces neuronal differentiation and cell death programs. (A) Representative live image of GB7 cells maintained in self-renewal conditions, 4 and 7 days after REST shRNA knockdown (shREST) and relative controls (CTRL: mock infected cells; NT shRNA: non targeting shRNA). REST knockdown causes reduction of proliferation and dramatic morphological changes, with appearance of a large proportion of flat and differentiated cells (arrows). (B) Colorimetric MTT-based cell viability assay performed on controls and shREST GB cell cultures. (C) Immunofluorescent analyses of controls and shREST self-renewing GB7 cells (7 days post infection). There is an evident reduction in the expression of neural progenitor (Nestin) and proliferation (P- HisH3) markers in shREST cells with respect to controls, with a parallel increase of neuronal differentiation (β3-tubulin) and apoptosis (Activated Caspase 3) markers. (D) Relative quantification of the numbers of immunoreactive cells in (C). CTRL: light gray bars; NT shRNA: dark gray bars; shREST: black bars. At least 700 cells per group were scored. (E) Detection of apoptosis on REST depleted GB cells. Seven days after siRNA transfection, the percentages of apoptotic GB7 cells in the cultures were analysed by FACS Annexin V–PE assay. (F) Luminometric detection of apoptosis on REST depleted GB cells. 48 and 72 hours after siRNA transfection, apoptosis in controls and siREST treated GB7 cells was analysed by Caspase-Glo 3/7 Assay. Data were analyzed by one-way ANOVA comparison of the controls groups and of the REST knockdown cultures. Results shown are relative to three independent experiments. Data are means ± s.d. *ns* not significant, *P<0.001, **P<0.05.

To uncover the functional role for REST in tumorigenic-competent GBM-derived cells, we assessed its ability to control self-renewal and neuronal differentiation, both programs known to be regulated by REST in normal NSCs [12], [13], [14], [15], [30], [31], [32]. REST targeting in GB cells by using lentiviral transduced short hairpin RNAs (shREST), resulted in a 75% silencing rate at the mRNA level (Figure S5A and B) and approx. 90% at the protein level (Figure S5C and D). In these cultures, REST targeting induced a marked morphological change in the cells, exhibiting a flatter appearance with respect to mock infected cells (CTRL) and cultures receiving non-targeting shRNA (NT shRNA) (Figure 3A). Furthermore, REST downregulation impaired self-renewal capacity, resulting in the inability to stably propagate shREST GB cells (Figure 3B and Figure S6). Similar effects were observed when performing a neurosphere assay on malignant GBM cells growing in suspension. In this assay, shREST cultures exhibited a decrease both in spheres’ number and size with respect to CTRL and NT shRNA cultures (Figure S7A-C). Antigenic analysis of the shREST cultures indicated a reduction of the number of nestin positive cells (CTRL: 90.9% ±12.4; NT shRNA: 91.9% ±9.7; shREST: 29.1% ±7.3; *P*<0.001) (Figure 3C and D). Of note, although the number of Olig2 positive cells did not substantially vary upon REST knockdown (*P*<0.05), we did observe a significant effect on the subcellular localization of Olig2 in nestin negative cells, where it exhibited a diffuse cytoplasmic and nuclear distribution, different from the typical nuclear-restricted expression (see arrows in Figure 3C). Concurrently, REST targeting induced a dramatic increase in the number of β3-tubulin positive cells (CTRL: 5.7% ±0.3; NT shRNA: 4.1% ±0.4; shREST: 76.0% ±9.5; *P*<0.001) (Figure 3C and D), as expected since it is among the REST-regulated genes, accompanied by a four-fold and ten-fold reduction of GFAP positive and phospho-H3 positive cells, respectively (*P*<0.001) (Figure 3C and D). Together these results indicated that REST knockdown induces cell-cycle arrest, impairs self-renewal and instructs the cells toward a neuronal fate. Similar results were obtained in three independent GB cell lines and in normal human NSCs and were further strengthened in GB cell cultures exposed to differentiating conditions (Figure S8). We also found that REST de-repression in GB cells stimulated an apoptotic program as shown by increased active-Caspase 3 immunoreactivity in REST-targeted cultures both in self-renewal (Figure 3C and D) and in differentiating conditions (Figure S8). To test the specificity of REST targeting, we transfected GB cells with REST-specific (siREST) or non-targeting (NT siRNA) siRNAs (Figure S9A and B). siREST transfection caused a relaxation of REST transcriptional repression activity on BDNF and SNAP25, two well-characterized REST-controlled genes (Figure S9C). Furthermore, siREST transfection resulted in increased apoptosis, as determined with Annexin V staining (CTRL: 36.3% ±3.5; NT siRNA: 38.4% ±4.7; siREST: 65.1% ±6.3; *P*<0.001) (Figure 3E) and luminometric assay for Caspases 3/7 activation (*P*<0.001) (Figure 3F).

### REST Knockdown Abolishes Tumor Formation by Human Tumorigenic-competent GBM Cells Xenograft and Impairs in vivo Tumor Growth of Established Xenograft Tumors

**Figure 4:**
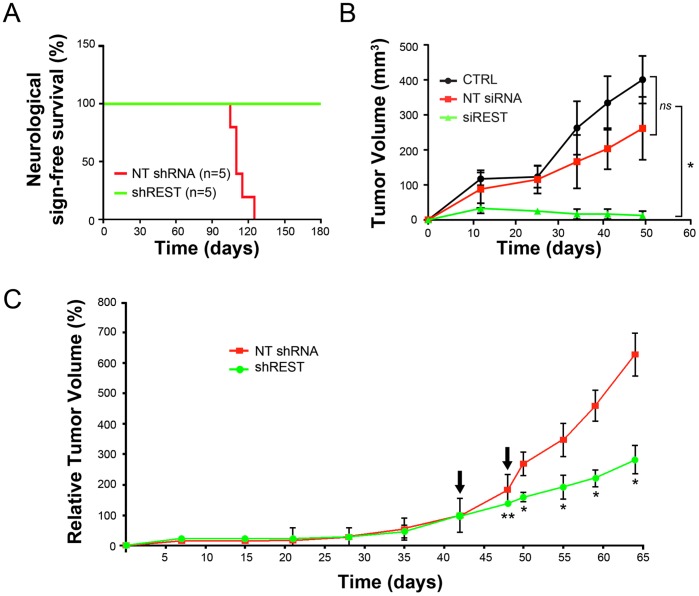
REST knockdown abolishes tumor formation by human tumorigenic-competent GBM cells xenograft and impairs *in vivo* tumor growth of established xenograft tumors. (A) Kaplan-Meier survival curve of SCID mice transplanted intracranially (*n = 4* per each group) with GB7 cells transduced with NT shRNA or shREST lentiviruses (prior to transplantation, cultures were subjected to three days of puromycin selection in order to eliminate non-infected cells). (B) REST siRNA targeting suppresses tumor formation of heterotopically GSC xenografts. Two days after GB7 cells transfection with non targeting (NT siRNA) and REST (siREST) siRNA were bilaterally injected subcutaneously in SCID mice (*n = 5* injected flanks per each group). An equal number of non-transfected cells were used as control (mock transfected cells: CTRL). Tumors formation was manifest after 12 days in the CTRL and NT siRNA groups while there was no evidence of tumor formation in siREST group. (C) *In vivo* intra-tumoral injection of REST shRNA impairs growth of heterotopic established tumors. GB7 cells were subcutaneously implanted into mice flanks and once tumors were established, lentiviral particles carrying either non-targeting shRNA (NT shRNA; *n = 4*) or shRNA directed against REST (shREST; *n = 4*) were delivered to tumor site through direct injection (two injections with 7 days interval; arrows in the graph). Tumor growth was unaffected in NT shRNA control group while a marked slowdown of tumor growth is evident in shREST group. Data are means ± s.d. *ns* not significant, *P<0.001, **P<0.01.

According to the *in vitro* requirements for REST in the survival and self-renewal capacity of GB cells demonstrated so far, it would be expected that delivery of REST-specific shRNA could affect their tumorigenic capability *in vivo*. To address this issue, GB cells were transduced with shREST or NT shRNA followed by a 3 days of puromycin selection to completely eliminate non transduced cells and afterwards cells were transplanted into the brains of immunocompromised SCID mice. Animals bearing shREST GB cells displayed no tumor formation and complete survival when compared to the ones receiving NT shRNA GB cells or not transduced GB cells (CTRL), up to 6 months post-transplantation, the latest time point we have examined (*P*<0.001) (Figure 4A). Histological analyses confirmed that NT shRNA transduced GB cells developed into tumors which invaded the brains, whereas no tumor developed in the brains receiving shREST transduced GB cells (Figure S10). To further confirm this result, mock transfected cells (CTRL) or NT siRNA or siREST transfected GB cells were heterotopically injected in immunocompromised SCID mice and tumor formation and growth was monitored. Within 12 days post-injection CTRL and NT siRNA injected animals developed tumor masses whose volume progressively increased with time, while in the same period no tumors could be detected in animals injected with REST targeted cells up to 50 days (*P*<0.001) (Figure 4B). However, these animals showed a delayed formation of tumors (80 days after the injection), likely because of the presence of unsuccessfully targeted cells. We conclude that targeting REST in GB cells is sufficient to impair their *in vivo* tumorigenic competence.

This was further verified by directly interfering with REST activity *in vivo*. For this purpose, NT shRNA or shREST lentiviral particles were directly delivered in GB-derived heterotopic tumors. Prior injection of lentiviral particles carrying eGFP reporter was used to determine the feasibility of the intratumoral delivery procedure and to set up the appropriate lentiviral injection dose (Figure S11A). Two intratumoral injections in seven days interval were performed in order to target a wider tumor area. In all experiments, animals receiving NT shRNA treatments exhibited the typical progressive growth of the tumor masses, whereas mice receiving shREST particles displayed a partial but significant reduction of the tumor growth rate, already after 10 days (day 50 after cell injection in Figure 4C) from the first lentiviral particles injection (*P*<0.001). Analysis of these tumor samples indicated the presence of areas exhibiting β3-tubulin and active Caspase 3 immunoreactivities, likely corresponding to the sites of shREST lentiviral particles injection (Figure S11B). Collectively, these data indicate that direct REST inhibition *in vivo* can interfere with growth of malignant GBM cell-derived xenograft tumors by activation of apoptosis and neuronal differentiation programs.

### REST Controls SCP1 and PTPN12 Phosphatases Expression Levels in Human Tumorigenic-competent GBM Cells by Modulating miR-124 Levels

Taken together, our study suggests that self-renewal, growth and survival of GBM-derived GB cells are critically dependent on REST activity and that inhibition of REST-regulated pathways might disclose potentially exploitable strategies towards GBM treatment in human. Nevertheless, our study reports the presence of REST positive cells in diverse gliomas and defines a role for REST as a positive regulator of self-renewal in tumorigenic-competent GBM-derived cells. Noteworthy, analyses of REST transcript in relation to the TCGA subgroups does not provide any hint about REST mRNA levels in relation to the TCGA subgroups. This is in agreement with very recent findings reporting that analysis of REST mRNA levels cannot be used to strictly create such a correlation due to a not linear stoichiometric relation between REST mRNA and protein levels [33]. Analysis of protein levels and distribution is extremely important in the case of REST as its post-translational modifications have been shown to regulate cell maintenance [30], [31]. Interfering with mechanisms regulating REST post-translational modifications have been shown to affect NSCs differentiation and cellular transformation in non-neural epithelial cells [20], [31]. In particular, the net balance between the β-TrCP-mediated ubiquitylation and the HAUSP-mediated deubiquitylation has been shown to control REST protein levels and determine cellular fate in mouse and human NSCs [30]. Of note, it has been reported that the association of TRF-2 with REST also prevents the ubiquitin proteasome-mediated degradation of REST [34], [35]. Consistently, our interrogation of the Human Protein ATLAS database indicates strong expression of HAUSP and TRF2 and, conversely, reduced level of expression of β-TrCP in malignant glioma specimens with respect to non-pathologic brain tissue (not shown).

**Figure 5:**
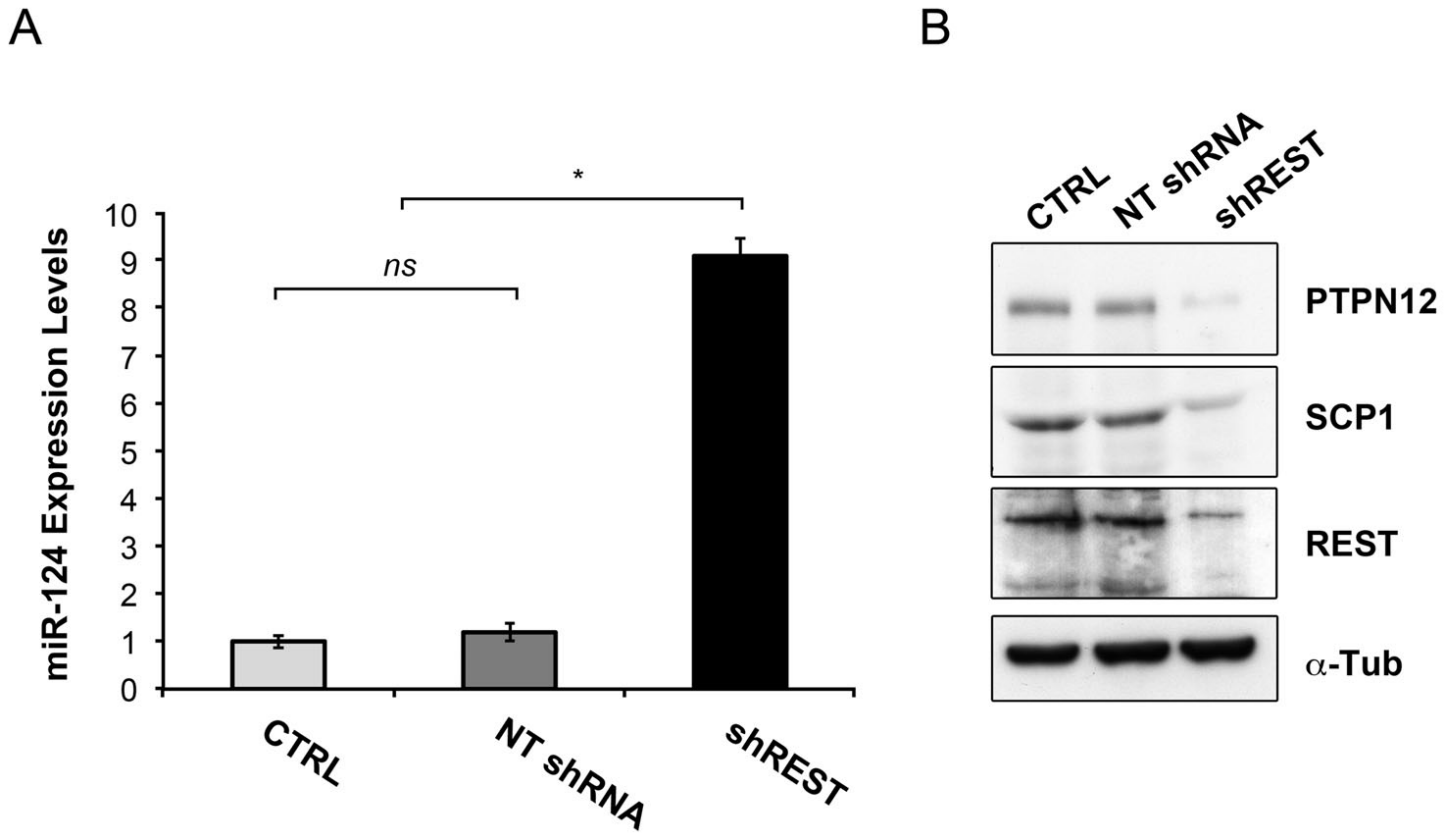
REST controls SCP1 and PTPN12 phosphatases expression levels in human tumorigenic-competent GBM cells by modulating miR-124 levels. (A) Quantitative RT-PCR analysis shows elevated miR-124 expression following REST knockdown in GB7 cells (shREST; 4 days post infection) compared to CTRL and NT shRNA control cultures. miR-124 expression level was normalized to RNU48 levels. Results are relative to three independent experiments. Data are means ± s.d. (B) Expression levels of miR-124 targets, SCP1 and PTPN12 phosphatases, in GB cells are controlled by REST. Downregulation of REST induces a reduction of the protein levels of SCP1 and PTPN12 in GB7 cells. Cultures were transduced with with non targeting (NT shRNA) and REST (shREST) shRNA, cultured for 4 days and analysed for expression of SCP1 and PTPN12 by western blot assay. CTRL sample represents the non trasfected culture. Alpha Tubulin levels were used as loading control. Data are means ± s.d. *ns* not significant, *P<0.001.

**Figure 6:**
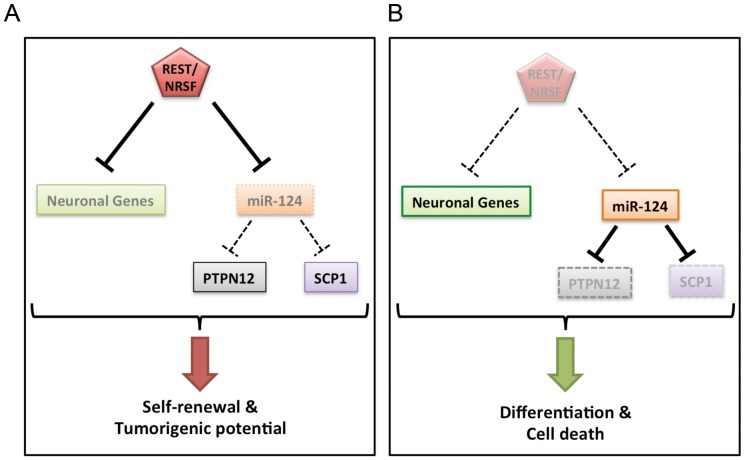
Schematic diagram illustrating one of the hypothetical regulatory mechanisms by which REST controls the cellular status in REST-expressing human tumorigenic-competent GBM cells (left box) and how this is perturbed in REST-depleted (right box) cells.

On the whole, our results support the hypothesis that in neural tissue, differently from non-neural compartments, REST favours the self-renewal and tumorigenic competence of tumor cells. The fact that REST can be either permissive or inhibitory for tumor formation, depending on tumor’s types, points to a context-specific modulation of REST-controlled pathways. miRNAs are crucial organizers of tissue-specificity [36] and REST has been shown to control the expression of different miRNAs [11], [37]. Among them, miR-124 represents the most differentially expressed miRNA between GBM and normal brain being poorly expressed in GBM [38], [39]. Of note, miR-124 levels directly correlated with patients’ survival [39]. miR-124 expression increases during NSC differentiation [40], and over-expression of miR-124 in putative GBM-derived stem cells has been shown to induce a dramatic increase in neuronal differentiation markers accompanied by reduced self-renewal and tumorigenicity [38]. We thus assayed miR-124 expression levels in normal and REST-depleted GB cells. Quantitative real-time PCR assay showed low miR-124 levels in CTRL and NT shRNA cultures, but a tenfold increase in shREST cultures (Figure 5A). We also found that the increase of miR-124 levels in REST-depleted GB cells resulted in a substantial reduction of SCP-1 and PTPN12 levels (Figure 5B), two phosphatases whose expression has been previously demonstrated to be controlled by a REST-miR-124 circuitry [41], [42]. Importantly, SCP-1 and PTPN12 play crucial roles in suppression of differentiative and oncogenic programs [42], respectively. These results indicated the conservation of REST inhibitory effect on miR-124 in GBM in human and we can speculate that one mechanism by which REST sustains self-renewal and tumorigenic competence of isolated GB cells might be through the repression of miR-124 expression (and deregulation of miR-124 targets) (Figure 6).

Our data define REST as an important mediator of GBM biology by demonstrating that REST is involved in GBM maintenance through regulation of the self-renewal, survival and differentiation of malignant GBM cells, and suggest that REST-regulated molecular circuitries may potentially turn into sensible targets for therapeutical intervention in GBM.

## Materials and Methods

### Ethics Statement

All the human tissues used in this study were collected only when necessary as part of treatment. Only samples in excess of what was needed for pathological assessment were used for research. Written informed consent to utilize excess tissue for research was obtained from each patient, and de-identified tissues, to protect anonymity, were used.

GB cell lines described in this manuscript were generated from tumor samples obtained during planned neurosurgical procedures for resection of newly diagnosed glioma at the Neurosurgical unit of the IRCCS Policlinico S. Matteo, Pavia, Italy. Surgery was performed according to the recommendation of the institutional review boards and in full agreement with the principles of the Declaration of Helsinki. For the use of the tumor samples for the generation of GB cell lines a written informed consent was obtained from each patient and the study approved by The Ethics Committees of the IRCCS Policlinico S. Matteo, Pavia, Italy. Cancer cell lines G144, G166 and G179, as well as the human NS (hNS; CB 541 and CB 192 cell lines) cells, were donated by Prof. Austin Smith (University of Cambridge, UK) who reported their generation and characterization elsewhere [29], [43]. GBM neurosphere line (NSGBnR1) were donated to our lab by Dr. Gaetano Finocchiaro (Istituto Neurologico C. Besta, Italy); these cells have been previously generated and described by the lab of Dr. Gaetano Finocchiaro [44]. Tumor specimens used for the immunohistochemistry analyses were collected in Italy at the Center of Policlinico di Monza Foundation and approval was obtained from the Institutional Ethics Board and in in full agreement with the principles of the Declaration of Helsinki.

All animal procedures were carried out in strict accordance with the legislation and guidelines for animal care and handling in Italy (DL116 92) approved by the Italian Ministry of Health, and those from the European Union (2003/65/CE from the European Parliament and Council July 2003). The protocols were approved by the Bioethical Committee of the University of Milan and by the Committee on the Ethics of Animal Experiments of the Fondazione IRCCS Istituto Nazionale dei Tumori. All experiments were designed to minimize the numbers of animals used and their discomfort.

### Tumor Samples and Cell Culture

The GB cell lines were established from fresh GBM surgical specimens collected directly from the operating theatre in PBS plus 0,6% glucose and immediately transported on ice to the cell culture room and processed and cultured as monolayer cultures as previously described [29]. Briefly, fresh tumor samples or xenografts were manually dissociated into single cells by enzymatic digestion with Accutase (Sigma) for 15–20 min at 37°C. Tumor cells suspension was then seeded into a fresh laminin-coated plastic plate. GB cells and control foetal human Neural Stem cells (hNS cells) were derived and grown in self renewal serum-free medium (SFM) containing EGF and FGF-2 (20 ng/ml; Peprotech), as previously described [29], [43]. Culture vessels were coated with 5 µg/ml Laminin (Invitrogen) for 3 hr prior to use. The phenotype of these cultures was confirmed by analysis of stem cell markers expression, *in vitro* assays of self-renewal, differentiation potential and *in vivo* tumor formation and propagation. In our analysis were included also other GB lines (G166 and G179 cells) and GBM neurospheres (NSGBnR1) previously described in other studies [29], [44]. For differentiation, single-cell suspensions of GB cells and normal hNS cells were seeded on Laminin-coated 48-well plates at 1.2×10^4^ cells/well density in proliferation medium and subjected to lentiviral transduction [29], [43]. 24 hours after infection, the cells were shifted in the same basal media deprived of both EGF and FGF-2 (growth factor withdrawal condition) and supplemented with a double amount of B27 supplement (4%) for 15 days. Half of the medium was replaced every 2–3 days along the differentiation period. GB7 and GB8 cell lines have been deposited in the cell repository at Biorep (Milan, Italy).

### REST Targeting in vitro

Knockdown of REST was achieved by means of lentiviral vector–mediated short hairpin RNA (shRNA) interference, using validated commercial lentiviral particles for the NRSF sequence (Santa Cruz Biotechnology), or through small interference RNA (siRNA), by employing oligonucleotides targeting human REST transcript sequence (Invitrogen). For the infection, lentiviral particles were gently diluted (1∶70) in proliferation medium supplemented with 5 µg/ml polybrene. Sub-confluent cultures were exposed to viral particles for 18–24 hours and then restored to classic compete medium. Knockdown efficiency was determined by immunocytochemistry, real time PCR and Western Blot analysis on GB infected cells. Control nontargeting shRNA (NT shRNA) Lentiviral Particles and cop GFP Control Lentiviral Particles (Santa Cruz Biotechnology) were used as controls. The latters were also used to monitor the transduction efficiency *in vitro* and *in vivo*. For REST- siRNA knockdown three different siRNAs, directed against different portion of human REST transcript, were characterized for their knockdown efficiency by real time PCR analysis on GB cells transfected with each REST targeting siRNAs or with universal negative scrambled controls (Invitrogen). The most efficient siRNA was used for all further experiments. Nucleofection technology (Amaxa, Lonza) was used to transfect the cells. For each nucleofection 4–5×10^6^ cells and 100pmole of siRNA were used following the protocol according to the manufacturer’s instruction. 50pmole of pMAX plasmid were used as control.

### Cell Assays

For colorimetric MTT-based cell viability assay cells were plated in triplicate into 24 well plate at a density of 2×10^4^ cells/well in self-renewal medium. At each time point considered, cells were exposed to 3-[4.5-dimethylthiazol-2-phenyl]-2.5-diphenyl-tertrazolium bromide (1 mg/ml) for 1 hour at 37°C and formazan release was quantified at 560 nm using a Microplate Reader (Biotek). For Active Caspase 3 analysis, 4×10^6^ cells were transfected with control and specific siRNA as indicated above and after 24 hours 1.5×10^4^ of nucleofected cells were plated in triplicate into 96 well plate in proliferation medium. Degree of apoptosis was assayed using Caspase-Glo® 3 Assay (Promega) on a Veritas™ Microplate Luminometer (Promega) according to the manufacturer’s protocol. For Annexin V staining, we used the Annexin V Apoptosis Detection Kit (BD Pharmingen™). Cells were nucleofected with NT siRNA and specific anti REST siRNA as indicated in the previous section and stained with Annexin V at different time points post nucleofection. The cells stained with Annexin V and relative controls were analyzed by cytofluorimetry with at least 10,000 events/determination, using FACSCantoII and DiVA software for acquisition and analysis (BD Pharmingen™).

### Mouse Xenograft Assay

Xenotransplantation experiments were performed on SCID mice (6–8 weeks old; Charles River Laboratories) housed in pathogen-free conditions. For s.c. inoculation SCID mice were anesthetized with i.m. ketamine and xylazine and a suspension 1∶1 of 0.5×10^6^ cells and Matrigel was injected bilaterally (200 µl/injection). Mice were monitored until the appearance of a mass and were sacrificed by CO_2_ asphyxiation when the tumors were in the exponential phase of growth. The explanted tumors were mechanically dissected into 2 mm fragments used for *in vitro* serial cell lines derivation and immunohistochemistry analysis. For *in vivo* REST knockdown analysis 10^7^ cells/mouse of viable GB cells non transfected and transfected with NT siRNA or the selected REST targeting siRNA were transplanted (48 hours after transfection procedure) into mice through s.c. injection following the same protocol described above. At least three SCID mice, receiving a bilateral s.c. cell inoculation for each experimental condition were used. For lentiviral-mediated REST shRNA treatment, GB cells were first s.c. injected to establish glioma xenografts as described above and the same quantity of NT lentiviral particles or REST targeting lentiviral particles were delivered to tumor sites through intratumoral injection. A second virus injection was performed one week after the first treatment. The lentiviral injection dose was defined by delivery of cop GFP Control Lentiviral Particles; 80 µl lentiviral particles in 300 µl PBS (i.e. 1.2–1.5×10^6^ viral infectious units was chosen as injection dose. For intracranial graft, SCID mice were anesthetized with i.m. ketamine and xylazine. Thereafter, animals received a stereotaxic injection of 1.5 µl (10^5^ cells/µl) of GB cells suspension in the left corpus striatum. Animals were monitored for 6 months for disease symptoms and were sacrificed when they showed weight loss or any other sign of disease.

### Statistical Analysis

Statistical significance was assayed using a Student’s unpaired *t* test or one-way ANOVA analysis of variance using the GraphPad Prism software. *P*<0.05 was considered statistically significant.

## Supporting Information

Figure S1 **REST expression is elevated in human gliomas.** (**A**) The median intensity of REST transcript expression is increased in human glioma patient samples in REMBRANDT (REpository for Molecular BRAin Neoplasia DaTa, National Cancer Institute) in comparison to non tumor tissue. (**B**) *REST* gene (4q12) deletion is associated with increased glioma patient survival in REMBRANDT.
(PDF)

Figure S2 **REST expression in non GBM human brain tumors.** (**A**) Grade I astrocytoma: positive nuclei are almost absent; (**B**) Grade II oligodendroglioma: ischemic neurons with intense nuclear staining; (**C**) Grade III oligodendroglioma: many nuclei are positive; (**D**) Pilocytic astrocytoma: all the nuclei are positive; (**E**) Positive nuclei in medulloblastoma with no neuronal differentiation; (**F**) negative nuclei in medulloblastoma with neuronal differentiation. DAB, 400×.
(PDF)

Figure S3 **Antigenic and biological properties of GB cells.** (**A**) Representative live image of GB7 cells in self-renewal conditions. (**B**) MTT cell viability assay on GB7 cells (OD%: relative optic density). Curve is relative to 2×10^4^ cells/well seeded on laminin-coated 24-Multiwell Plate. Curve points are shown as mean ± s.d. of three independent replicates. (**C**) GB7 cells show an antigenic expression pattern comparable to adherent human fetal neural stem cell markers (NSCs), with high expression of NSC markers (Nestin, Sox2, Olig2, Vimentin) and negligible expression of neuronal (β3-tubulin) or glial (GFAP) markers. (**D**) Orthotopically xenografted GB7 cells form tumors in SCID mice. Kaplan-Meyer survival curve of SCID mice (*n* = 5) transplanted intracranially with GB7 cells (150,000 cells per injection).
(PDF)

Figure S4 **Elevated REST expression levels are preserved in serial heterotopic GB7 cell-derived xenografts and in serial GB7 cell lines.** (**A**) Immunohistochemistry for REST (Left) and Nestin (Right) in tumors derived from GB7 cells serial heterotopic xenografts (representative images from second serial xenografts). (**B**) GB cells can be stably re-derived following serial heterotopic xenograft propagation. After four *in vivo* passages GB cells maintain the same morphological and antigenic features of the parental GB cell line. **Left:** Representative live images of proliferating GB7 serial cell lines I (derived from first xenograft), II (derived from first serial xenograft), III (derived from second serial xenograft) and IV (derived from third serial xenograft). **Right:** Antigenic characterization for neural progenitors markers (nestin and Sox2) of GB7 serial cell lines I, II, III and IV. (**C**) Quantitative Real Time PCR analysis showing REST expression in serial GB7 cell lines. In all serial GB7 cell lines REST expression is grossly maintained (GB7 I and IV cells: *P*<0.001; GB7 II and III cells: *P*<0.05) to levels similar to GB7 parental cells. REST expression levels were normalized to GAPDH. Data are relative to three independent experiments and are presented as means ± s.d. (**D**) REST immunofluorescent staining on serial GB7 cell lines. Similarly the parental GB7 cells, all the serial GB7 cell lines homogenously show a prominent REST nuclear immunoreactive signal.
(PDF)

Figure S5 **REST shRNA silencing efficiency in GB7 cells.** (**A**) Live phase contrast and fluorescence images of GB7 cells 72 hours after infection (infection time: 24 hours) with GFP-expressing lentiviral particles to assess infection efficiency. At this time point, a 77.5% ±6.7 efficiency of infection was determined. (**B, C and D**) GB7 cells infected with lentiviral particles carrying anti-REST shRNA (shREST) show a strong reduction in REST expression both at mRNA and protein levels as determined by (**B**) quantitative Real Time PCR analysis (REST transcript level in CTRL group is set as 100; NT shRNA: 91.4% ±7.4; shREST: 27.3% ±6.6, *P*<0.001), (**C**) Western blotting (a 88.9% reduction od REST immunoreactive signal in shREST group with respect to control groups is determined) and (**D**) immunofluorescent staining. No effects are evident on REST levels following infection of GB7 cells with non-targeting control shRNA (NT shRNA: 91.3% ±8.7, *P* not significant) or control GFP-expressing (eGFP: 94.1% ±5.7, *P* not significant) lentiviral particles. CTRL group is represented by mock infected cultures. The immunofluorescent staining in (**D**) shows a marked reduction in nuclear REST immunoreactivity with some degree of persistence of signal in small dots inside the nucleus. Results shown are relative to three independent experiments. Data are means ± s.d.
(PDF)

Figure S6 **REST silencing in human tumorigenic-competent GBM cells is not permissive for self-renewal.** GB7 cells 48 hours after infection (infection time: 24 hours) with non-targeting control shRNA (NT shRNA) or control GFP-expressing (eGFP) lentiviral particles or with lentiviral particles carrying shRNA anti-REST (shREST) were exposed to puromycin selection (lentiviral particles carry a puromycin selection cassette) in order to remove the fraction of non-infected cells. CTRL group is represented by mock-infected cultures. Pictures are relative to the different experimental groups at 0, 4, 7 and 12 days of puromycin selection. Cultures which integrate controls lentiviral particles (NT shRNA and eGFP groups) readily expand in puromycin selection; shREST cells, even surviving, never proliferate and degenerate after two weeks of selection. Non-infected cultures (CTRL) were readily killed by puromycin selection. Results shown are representative of three independent experiments.
(PDF)

Figure S7 **REST knockdown in human tumorigenic-competent GBM cells impairs sphere formation efficiency and sphere size.** (**A**) 24 hours after infection, tumorigenic-competent GBM cells grown as neurosphere (NSGBnR1 line) infected with non-targeting control shRNA (NT shRNA) or with lentiviral particles carrying shRNA anti-REST (shREST) were plated (10^4^ cells per well in a 24 well plate). CTRL group is represented by mock-infected cultures. 24 hours after plating, cells were exposed to 1 µm/mL puromycin selection (lentiviral particles carry a puromycin selection cassette) in order to remove the fraction of non-infected cells (no puromycin selection was performed on CTRL cells). Representative live image of cells ten days after plating show that the shREST cells formed fewer and smaller neurospheres in comparison to both CTRL and NT shRNA groups. Results shown are relative to three independent experiments. (**B and C**) 24 hours after infection, NSGBnR1 neurosphere cells infected with non-targeting control shRNA (NT shRNA) or with lentiviral particles carrying shRNA anti-REST (shREST) were plated (1 cells per well in a 96 well plate; three plates for a total of 288 wells were plated per each experimental group). CTRL group is represented by mock-infected cultures. Differently from cultures shown in (A), cells were not exposed to puromycin selection in order to avoid any possible interference with single cell analysis. The presence of clonal sphere in each well was determined in two weeks. Quantification shows reduced sphere number (total number of spheres in CTRL group is set as 100; NT shRNA: 98.3% ±14.8, *P* not significant; shREST: 14.0% ±5.8 *P*<0.001) (**B**) and size (sphere diameter) (**C**) for shREST cells with respect to control groups. Data are means ± s.d. (*n* = 3). *ns* not significant, *P<0.001**,** **P<0.05.
(PDF)

Figure S8 **REST knockdown triggers neuronal differentiation and cell death programs in human tumorigenic-competent GBM cells.** (**A**) Representative live image of GB7 cells exposed to differentiating conditions for 7 and 14 days after lentiviral REST shRNA knockdown (shREST) and relative controls (CTRL: untreated; NT shRNA: non targeting shRNA). REST knockdown produces drop of proliferation and dramatic morphological changes, with appearance of a pronounced proportion of flat and differentiated cells. (**B**) Immunofluorescent analyses of control(s) and shREST GB7 cells in differentiation contitions (14 days post lentiviral shRNA infection). The number of cells immunopositive for neural progenitor (Nestin and Olig2) and proliferation (P-HisH3) markers in shREST cultures is strongly lessened with respect to controls (CTRL and NT shRNA groups), with a parallel increase of neuronal differentiation (β3-tubulin) and apoptosis (Activated Caspase 3) markers. The number of GFAP expressing cells is reduced in shREST cultures with respect to the control cultures, indicating that REST derepression strongly favors the conversion toward the neuronal lineage at the expenses of the glial lineage. (**C**) Relative quantification of the numbers of immunoreactive cells in (B). CTRL: light gray bars; NT shRNA: dark gray bars; shREST: black bars. At least 700 cells per group were scored. Results shown are relative to three independent experiments. Data are means ± s.d. *ns* not significant, *P<0.001**,** **P<0.05.
(PDF)

Figure S9 **REST siRNA silencing efficiency in GB cells.** (**A**) Live fluorescent images of GB7 cells 48 hours after nucleofection with three different anti REST siRNAs (siREST#1, siREST#2, siREST#3) each directed against a distinct region of REST transcript or with non targeting control siRNA (NT siRNA) and co-transfected with a GFP-carrying plasmid (eGFP) to assess nucleofection efficiency. The transfection efficiency and the fluorescence levels on siREST cultures are comparable to control cultures. Average nucleofection efficiency: 74.6% ±8.3. (**B**) REST siRNA targeting efficiently reduces REST transcript levels in GB cells. Real Time PCR analysis showing REST expression in siREST nucleofected GB7 cells and in control (CTRL, eGFP and NT siRNA) cultures. REST expression level was normalized to GAPDH. REST transcript level in CTRL group is considered as 100%. Best knockdown efficiency is achieved with siREST#1 (siREST#1: 17.3% ±3.5, *P*<0.001; siREST#2: 29.7% ±5.1, *P*<0.001; siREST#3: 24.2% ±4.4, *P*<0.001) which was selected for further experiments. No effect on REST levels after nucleofection in NT siRNA (116.4±15.6, *P* not significant) or GFP (98.3±17.1, *P* not significant) cultures. Data are relative of three independent experiments and are presented as mean ± s.d. (**C**) REST knockdown de-represses the REST-mediated silencing activity on target genes transcription in GB cells. Quantitative Real Time PCR analysis of transcripts levels of REST-controlled genes (BDNF and SNAP25) in siREST nucleofected (96 hours after transfection) and control GB7 cultures. RNA was purified from GB7 cells nucleofected with either anti-REST siRNA #1 (siREST#1) or non-targeting control siRNA (NT siRNA). RNA extracted from mock nucleofected cells was used as control (CTRL). GAPDH was used as housekeeping gene to normalize REST expression levels. The mRNA expression levels of CTRL group was set to 100 for each gene and data are shown as percentage of CTRL. REST targeting leads to a marked de-repression of REST-target genes (BDNF: 224.4% ±17.2, *P*<0.001; SNAP25: 526.9% ±17.8, *P*<0.001). No effect on expression levels of all the genes after transfection with NT siRNA (BDNF: 77.8±14.4, *P*>0.001; SNAP25: 74.6% ±18.9, *P*>0.001). Data are relative of three independent experiments and are presented as means ± s.d.
(PDF)

Figure S10 **REST knockdown abolishes orthotopic xenograft tumor formation by human tumorigenic-competent GBM cells.** GB7 cells transduced with NT shRNA or shREST lentiviral particles were injected into brains of SCID mice (150,000 cells per mouse; prior to transplantation, cultures were puromycin-selected for three days in order to eliminate non-infected cells). Four mice were injected for each group. Mice in the control group were sacrificed upon the development of neurologic signs. All the mice bearing shREST GB7 cells did not develop neurologic signs and were sacrificed after 180 days without evidence of tumor formation. (**A** and **B**) Representative images of coronal sections of grafted brains. H&E staining demonstrated the presence of brain tumors in mice injected with NT shRNA GB7 cells (**A**), while no tumors were observed in brains of mice injected with shREST GB7 cells (**B**). Insets in (A and B) show whole sections of the grafted brains. (**C–F**) Immunohistochemical staining of brain sections from NT shRNA mice (**C** and **E**) and shREST mice (**D** and **F**) with antibodies against nestin (**C** and **D**) and PCNA (**E** and **F**).
(PDF)

Figure S11 ***In vivo*** **intra-tumoral injection of REST shRNA impairs growth of heterotopic established tumors.** (**A**) *In vivo* lentiviral injection of control GFP-expressing lentiviral particles in established GB7 cells-derived xenograft tumors in SCID mice. GB7 cells were subcutaneously implanted into mice flanks and, once tumors were well established (42 days after grafting), GFP-expressing lentiviral particles were delivered to the tumor site through direct single injection in order to assess the infection efficiency. One-week post virus injection mice were sacrificed and tumor tissue sectioned. Representative fluorescent images of tumor section show the presence of eGFP signal indicative for the occurrence of a local infection of the tumor mass. (**B**) Lentiviral particles carrying either non-targeting shRNA or shRNA directed against REST were delivered to tumor site (42 days old GB7 cell-derived xenografts; *n = 4* for each group) through direct injection (two injections with 7 days interval). Fifteen days after the last virus injection, mice were sacrificed and tumor tissue sectioned. H&E staining of tumor tissue sections enlightens the presence of areas with reduced cellular density in the shREST injected tumors, indicating focal infected areas where possibly cell death has occurred. The tissue from NT shRNA infected tumors shows a more uniform cellular density. Representative immunohistochemical staining of tumor tissue sections from NT shRNA and shREST groups with antibodies against β3-tubulin and Active Caspase-3 show indeed the presence of immunoreactive areas in shREST infected tumors.
(PDF)
