# Correction: REST Controls Self-Renewal and Tumorigenic Competence of Human Glioblastoma Cells

**DOI:** 10.1371/journal.pone.0139645

**Published:** 2015-09-25

**Authors:** The PLOS ONE Staff

There are errors in the Funding section. The publisher apologizes for the errors. The correct funding information is as follows: This work was supported by NeuroScreen (FP6, European Union Health-2007-B-222943) and Progetto Giovani Ricercatori (Italian Ministry of Health, GR-2008-1146615) to Luciano Conti and by Progetto Oncologico “Characterization of the molecular mechanisms regulating brain tumors growth and chemioresistance” (Italian Ministry of Health, 2008–2011) and partially by NeuroStemcell (European Community’s Seventh Framework Programme grant agreement nr.222943) to EC. The funders had no role in study design, data collection and analysis, decision to publish, or preparation of the manuscript.
