# Supplementary material for: REST Controls Self-Renewal and Tumorigenic Competence of Human Glioblastoma Cells
Source: PLoS One. 2012 Jun 11;7(6):e38486. doi: 10.1371/journal.pone.0038486 (PMC3372516; doi:10.1371/journal.pone.0038486)
Supplement: Figure S1 — REST expression is elevated in human gliomas. (A) The median intensity of REST transcript expression is increased in human glioma patient samples in REMBRANDT (REpository for Molecular BRAin Neoplasia DaTa, National Cancer Institute) in comparison to non tumor tissue. (B) REST gene (4q12) deletion is associated with increased glioma patient survival in REMBRANDT. (PDF) [file pone.0038486.s001.pdf]

A

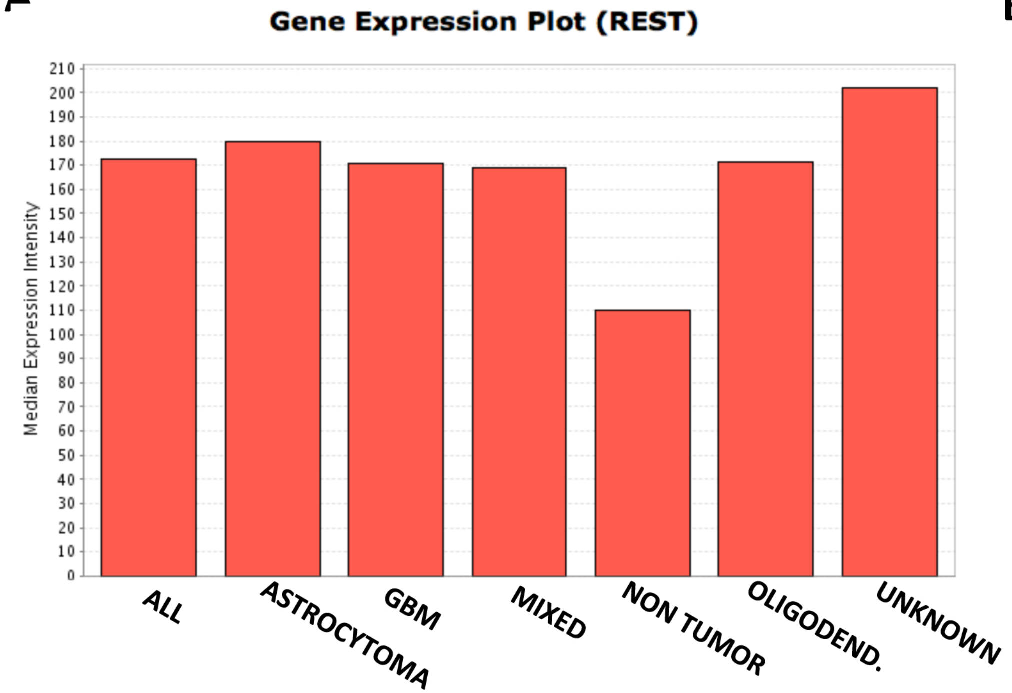

B

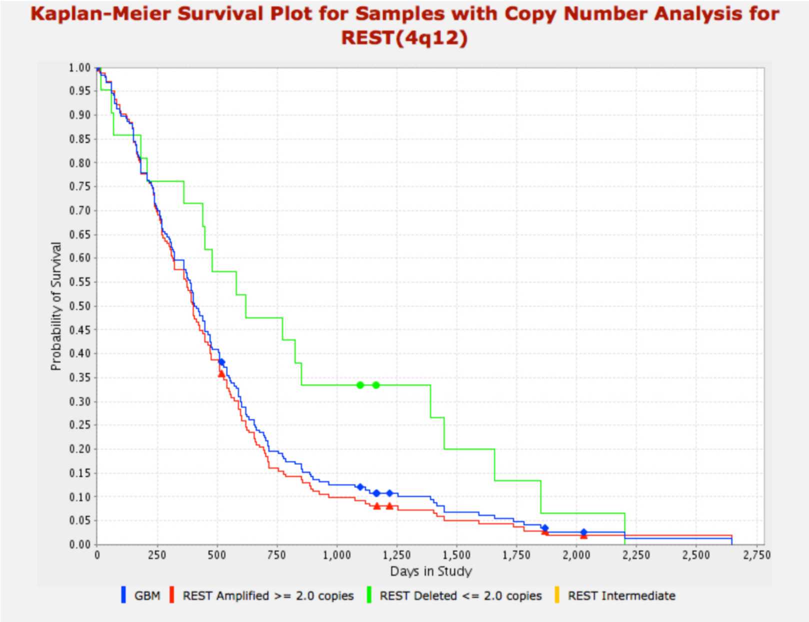

Conti et al., Figure S1
