# Supplementary figures and images for: REST Controls Self-Renewal and Tumorigenic Competence of Human Glioblastoma Cells
Source: PLoS One. 2012 Jun 11;7(6):e38486. doi: 10.1371/journal.pone.0038486 (PMC3372516; doi:10.1371/journal.pone.0038486)

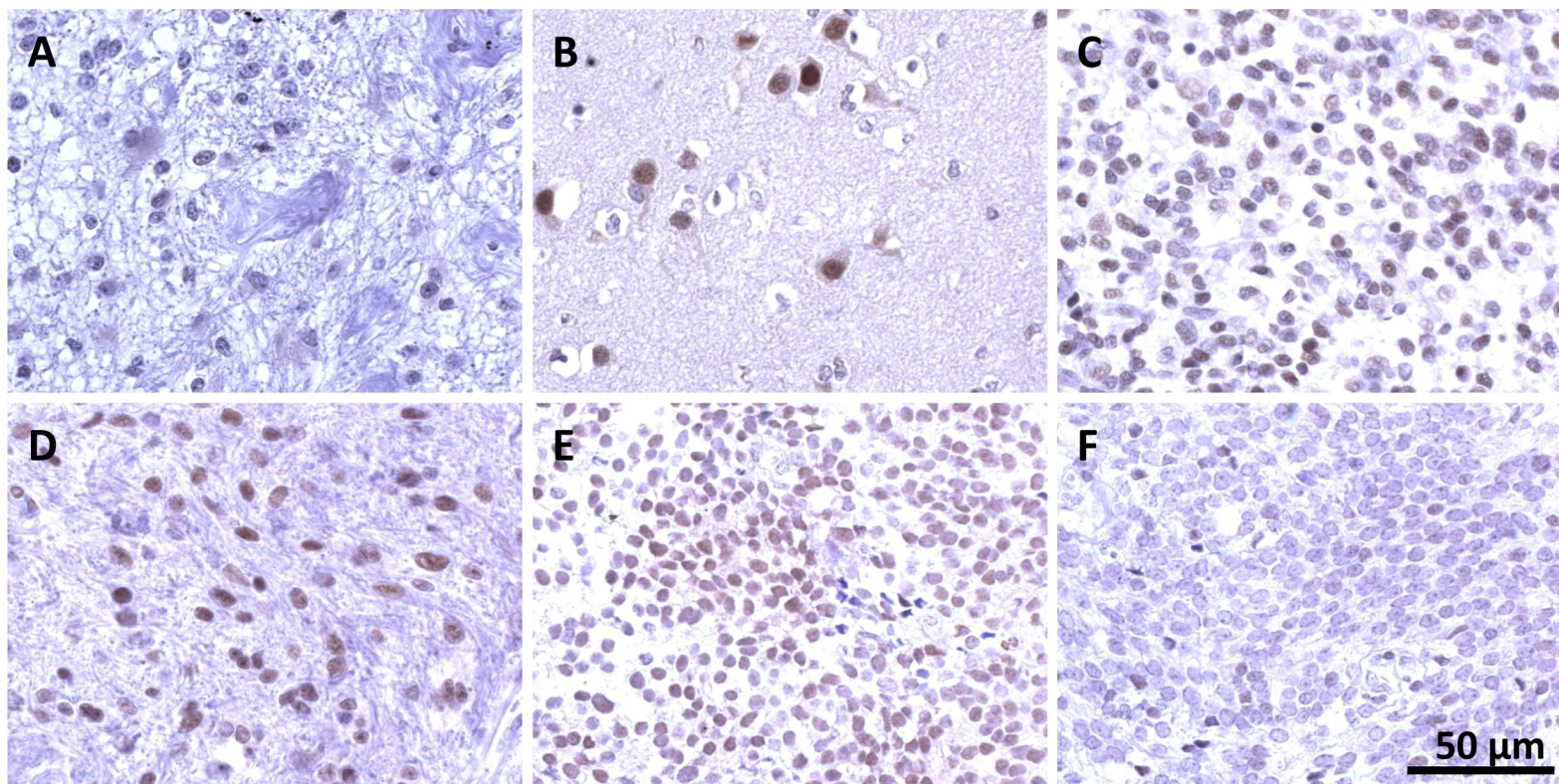

Supplement: Figure S2 — REST expression in non GBM human brain tumors. (A) Grade I astrocytoma: positive nuclei are almost absent; (B) Grade II oligodendroglioma: ischemic neurons with intense nuclear staining; (C) Grade III oligodendroglioma: many nuclei are positive; (D) Pilocytic astrocytoma: all the nuclei are positive; (E) Positive nuclei in medulloblastoma with no neuronal differentiation; (F) negative nuclei in medulloblastoma with neuronal differentiation. DAB, 400×. (PDF) [file pone.0038486.s002.pdf]

**A**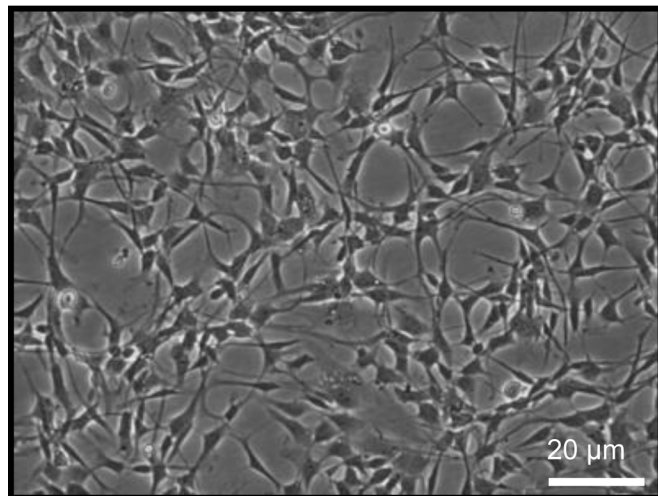**B**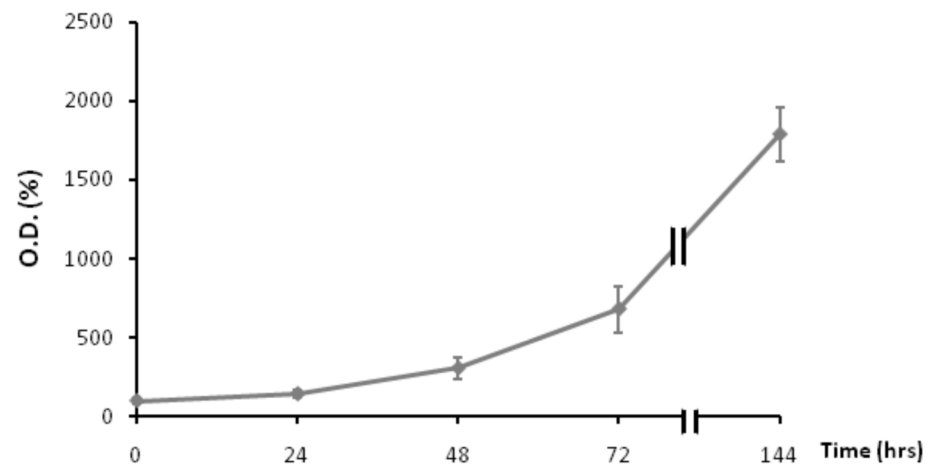**C**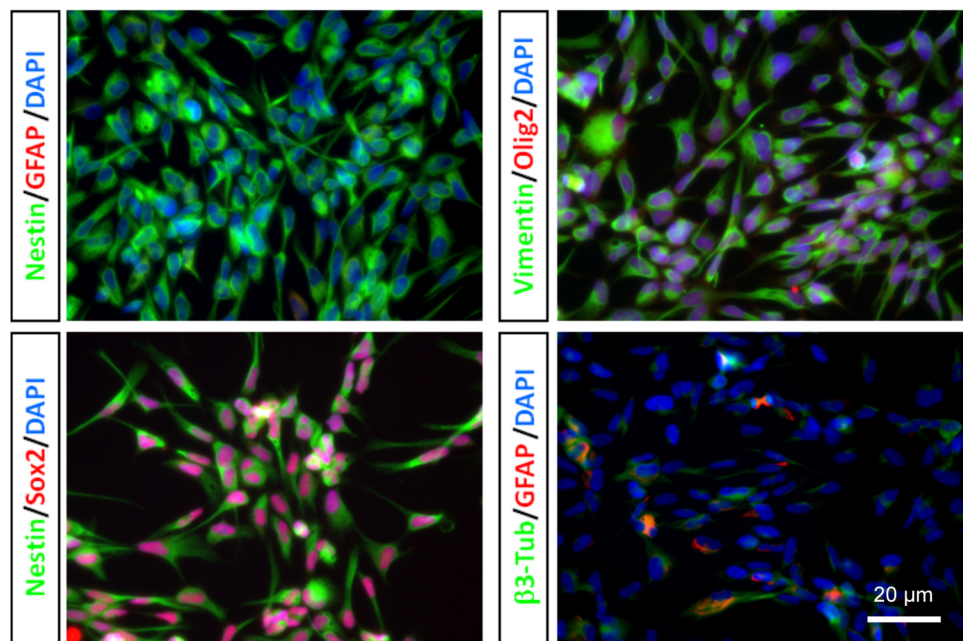**D**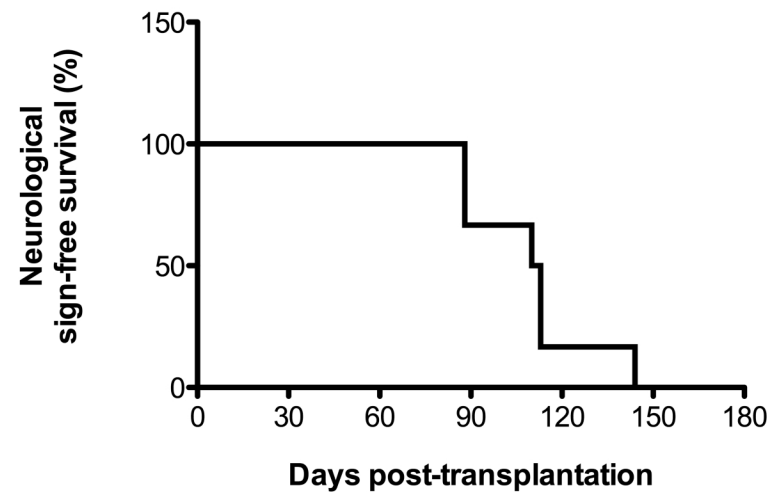

Supplement: Figure S3 — Antigenic and biological properties of GB cells. (A) Representative live image of GB7 cells in self-renewal conditions. (B) MTT cell viability assay on GB7 cells (OD%: relative optic density). Curve is relative to 2×10^4^ cells/well seeded on laminin-coated 24-Multiwell Plate. Curve points are shown as mean ± s.d. of three independent replicates. (C) GB7 cells show an antigenic expression pattern comparable to adherent human fetal neural stem cell markers (NSCs), with high expression of NSC markers (Nestin, Sox2, Olig2, Vimentin) and negligible expression of neuronal (β3-tubulin) or glial (GFAP) markers. (D) Orthotopically xenografted GB7 cells form tumors in SCID mice. Kaplan-Meyer survival curve of SCID mice (n = 5) transplanted intracranially with GB7 cells (150,000 cells per injection). (PDF) [file pone.0038486.s003.pdf]

**A**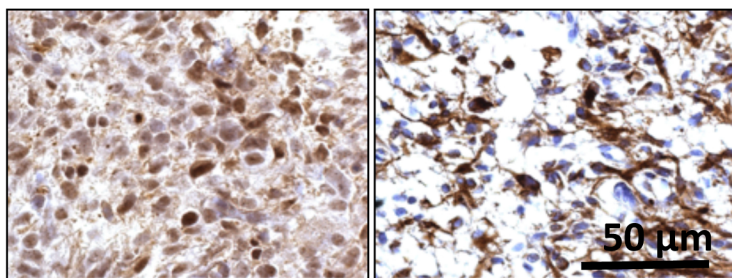**C**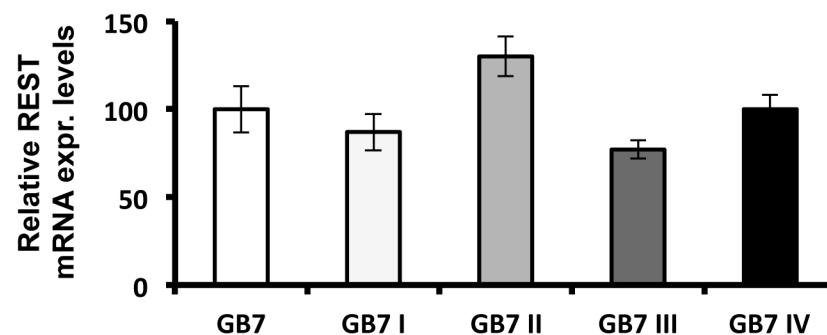**B**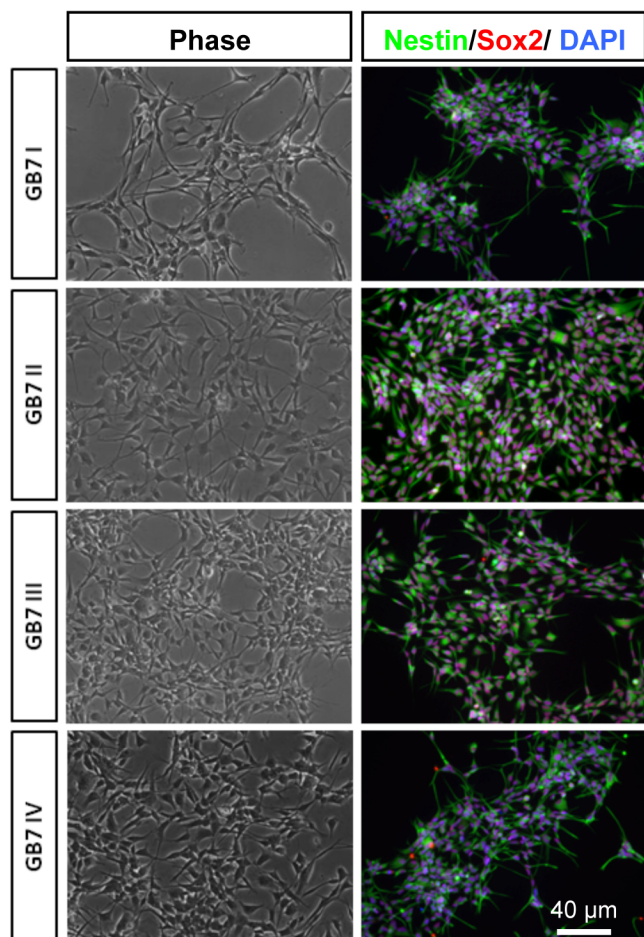**D**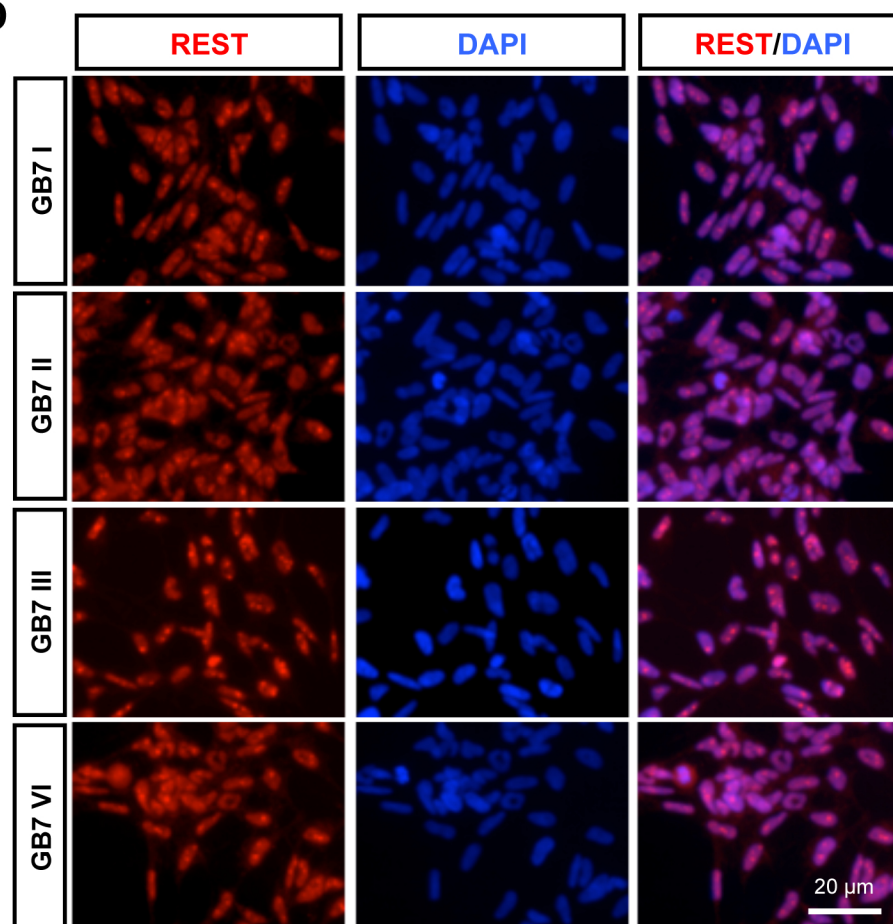

Supplement: Figure S4 — Elevated REST expression levels are preserved in serial heterotopic GB7 cell-derived xenografts and in serial GB7 cell lines. (A) Immunohistochemistry for REST (Left) and Nestin (Right) in tumors derived from GB7 cells serial heterotopic xenografts (representative images from second serial xenografts). (B) GB cells can be stably re-derived following serial heterotopic xenograft propagation. After four in vivo passages GB cells maintain the same morphological and antigenic features of the parental GB cell line. Left: Representative live images of proliferating GB7 serial cell lines I (derived from first xenograft), II (derived from first serial xenograft), III (derived from second serial xenograft) and IV (derived from third serial xenograft). Right: Antigenic characterization for neural progenitors markers (nestin and Sox2) of GB7 serial cell lines I, II, III and IV. (C) Quantitative Real Time PCR analysis showing REST expression in serial GB7 cell lines. In all serial GB7 cell lines REST expression is grossly maintained (GB7 I and IV cells: P<0.001; GB7 II and III cells: P<0.05) to levels similar to GB7 parental cells. REST expression levels were normalized to GAPDH. Data are relative to three independent experiments and are presented as means ± s.d. (D) REST immunofluorescent staining on serial GB7 cell lines. Similarly the parental GB7 cells, all the serial GB7 cell lines homogenously show a prominent REST nuclear immunoreactive signal. (PDF) [file pone.0038486.s004.pdf]

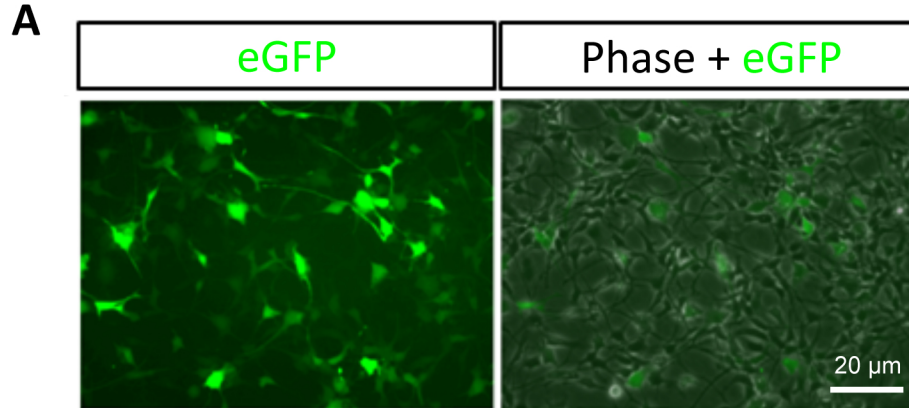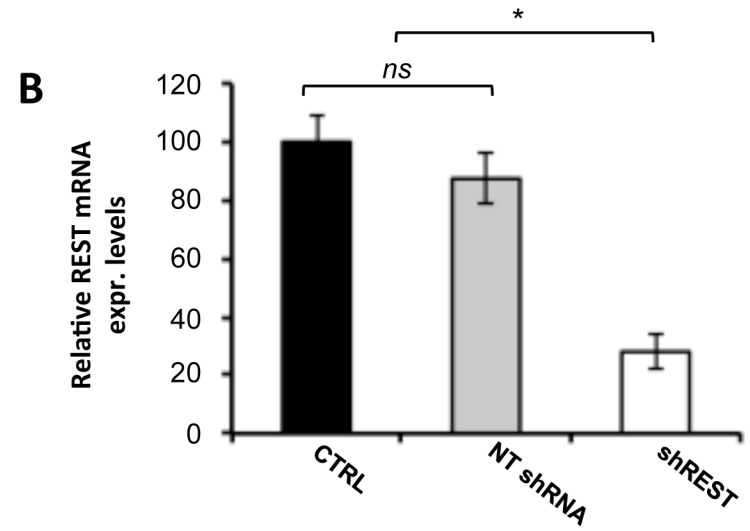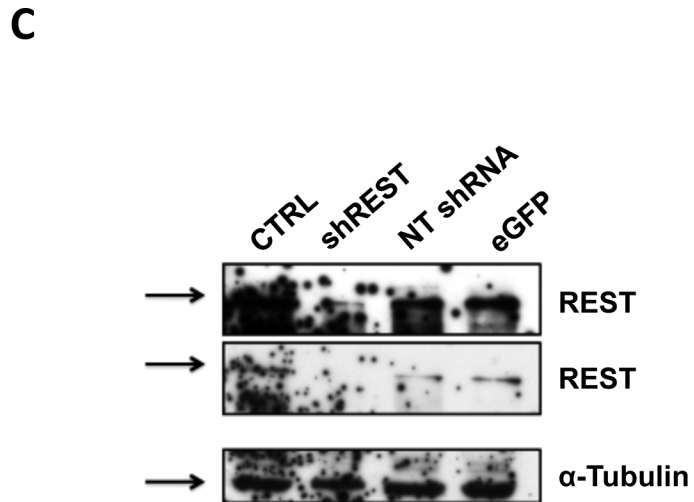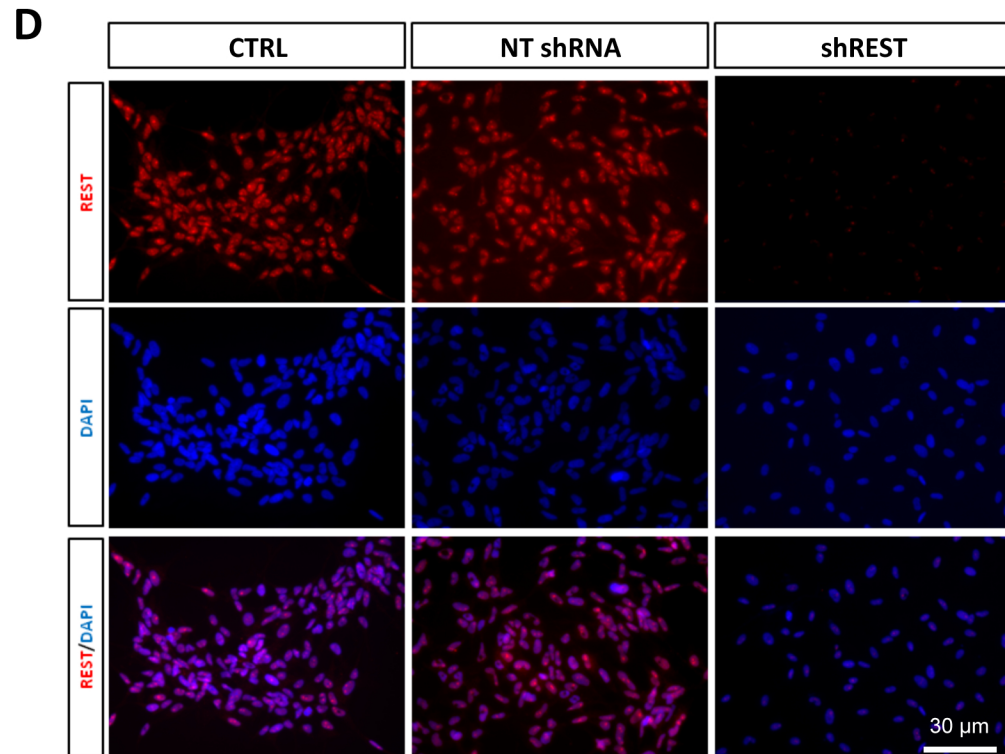

Supplement: Figure S5 — REST shRNA silencing efficiency in GB7 cells. (A) Live phase contrast and fluorescence images of GB7 cells 72 hours after infection (infection time: 24 hours) with GFP-expressing lentiviral particles to assess infection efficiency. At this time point, a 77.5% ±6.7 efficiency of infection was determined. (B, C and D) GB7 cells infected with lentiviral particles carrying anti-REST shRNA (shREST) show a strong reduction in REST expression both at mRNA and protein levels as determined by (B) quantitative Real Time PCR analysis (REST transcript level in CTRL group is set as 100; NT shRNA: 91.4% ±7.4; shREST: 27.3% ±6.6, P<0.001), (C) Western blotting (a 88.9% reduction od REST immunoreactive signal in shREST group with respect to control groups is determined) and (D) immunofluorescent staining. No effects are evident on REST levels following infection of GB7 cells with non-targeting control shRNA (NT shRNA: 91.3% ±8.7, P not significant) or control GFP-expressing (eGFP: 94.1% ±5.7, P not significant) lentiviral particles. CTRL group is represented by mock infected cultures. The immunofluorescent staining in (D) shows a marked reduction in nuclear REST immunoreactivity with some degree of persistence of signal in small dots inside the nucleus. Results shown are relative to three independent experiments. Data are means ± s.d. (PDF) [file pone.0038486.s005.pdf]

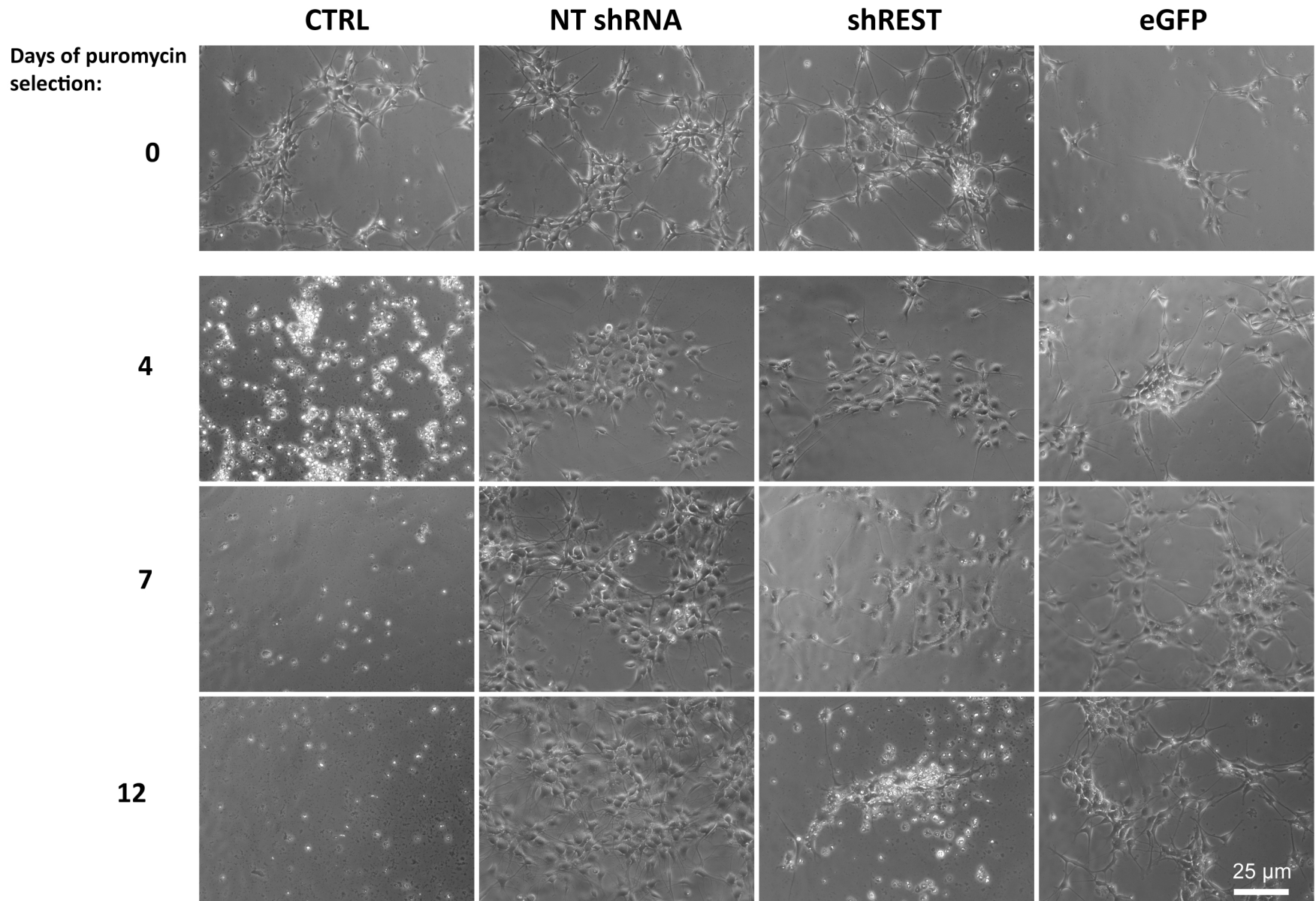

Supplement: Figure S6 — REST silencing in human tumorigenic-competent GBM cells is not permissive for self-renewal. GB7 cells 48 hours after infection (infection time: 24 hours) with non-targeting control shRNA (NT shRNA) or control GFP-expressing (eGFP) lentiviral particles or with lentiviral particles carrying shRNA anti-REST (shREST) were exposed to puromycin selection (lentiviral particles carry a puromycin selection cassette) in order to remove the fraction of non-infected cells. CTRL group is represented by mock-infected cultures. Pictures are relative to the different experimental groups at 0, 4, 7 and 12 days of puromycin selection. Cultures which integrate controls lentiviral particles (NT shRNA and eGFP groups) readily expand in puromycin selection; shREST cells, even surviving, never proliferate and degenerate after two weeks of selection. Non-infected cultures (CTRL) were readily killed by puromycin selection. Results shown are representative of three independent experiments. (PDF) [file pone.0038486.s006.pdf]

**A**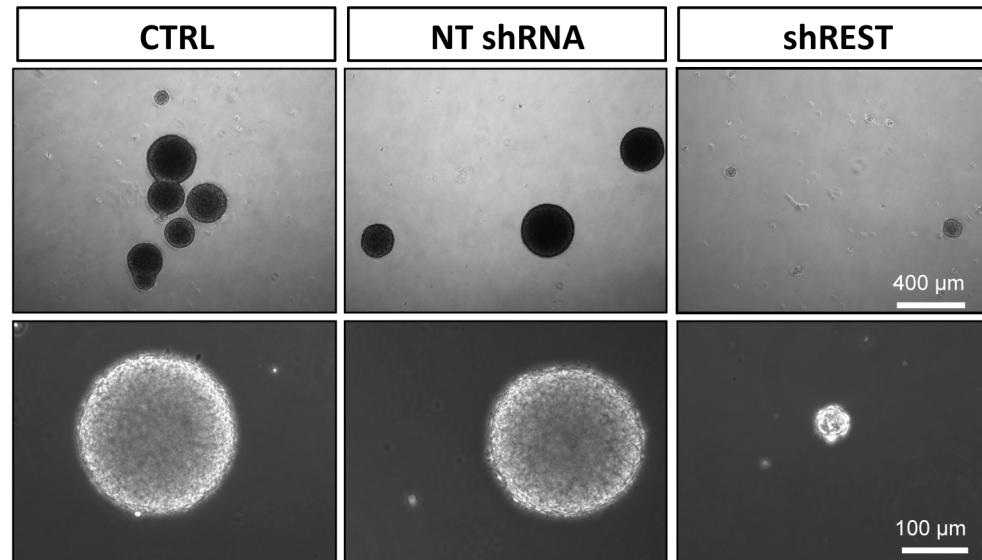**B**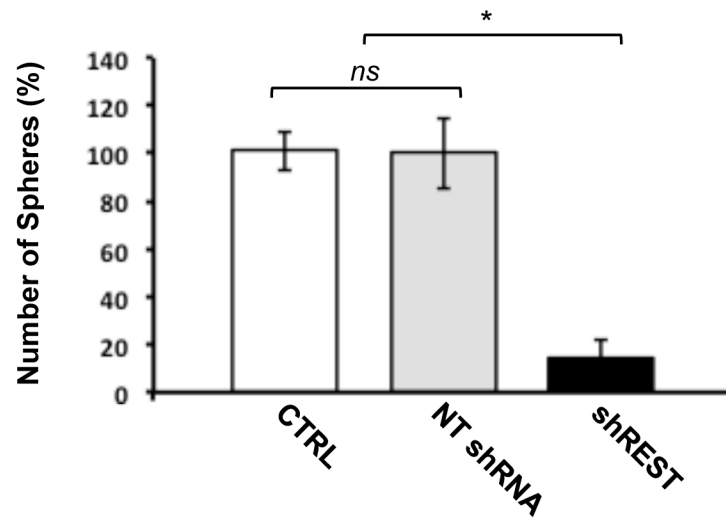**C**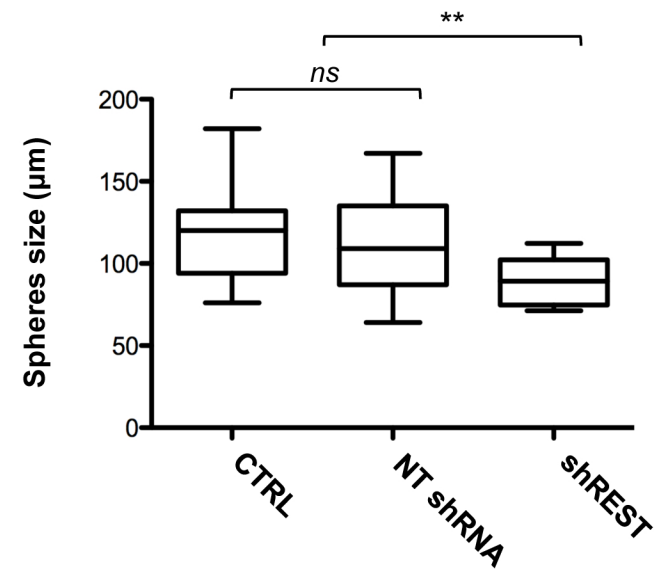

Supplement: Figure S7 — REST knockdown in human tumorigenic-competent GBM cells impairs sphere formation efficiency and sphere size. (A) 24 hours after infection, tumorigenic-competent GBM cells grown as neurosphere (NSGBnR1 line) infected with non-targeting control shRNA (NT shRNA) or with lentiviral particles carrying shRNA anti-REST (shREST) were plated (104 cells per well in a 24 well plate). CTRL group is represented by mock-infected cultures. 24 hours after plating, cells were exposed to 1 µm/mL puromycin selection (lentiviral particles carry a puromycin selection cassette) in order to remove the fraction of non-infected cells (no puromycin selection was performed on CTRL cells). Representative live image of cells ten days after plating show that the shREST cells formed fewer and smaller neurospheres in comparison to both CTRL and NT shRNA groups. Results shown are relative to three independent experiments. (B and C) 24 hours after infection, NSGBnR1 neurosphere cells infected with non-targeting control shRNA (NT shRNA) or with lentiviral particles carrying shRNA anti-REST (shREST) were plated (1 cells per well in a 96 well plate; three plates for a total of 288 wells were plated per each experimental group). CTRL group is represented by mock-infected cultures. Differently from cultures shown in (A), cells were not exposed to puromycin selection in order to avoid any possible interference with single cell analysis. The presence of clonal sphere in each well was determined in two weeks. Quantification shows reduced sphere number (total number of spheres in CTRL group is set as 100; NT shRNA: 98.3% ±14.8, P not significant; shREST: 14.0% ±5.8 P<0.001) (B) and size (sphere diameter) (C) for shREST cells with respect to control groups. Data are means ± s.d. (n = 3). ns not significant, *P<0.001, **P<0.05. (PDF) [file pone.0038486.s007.pdf]

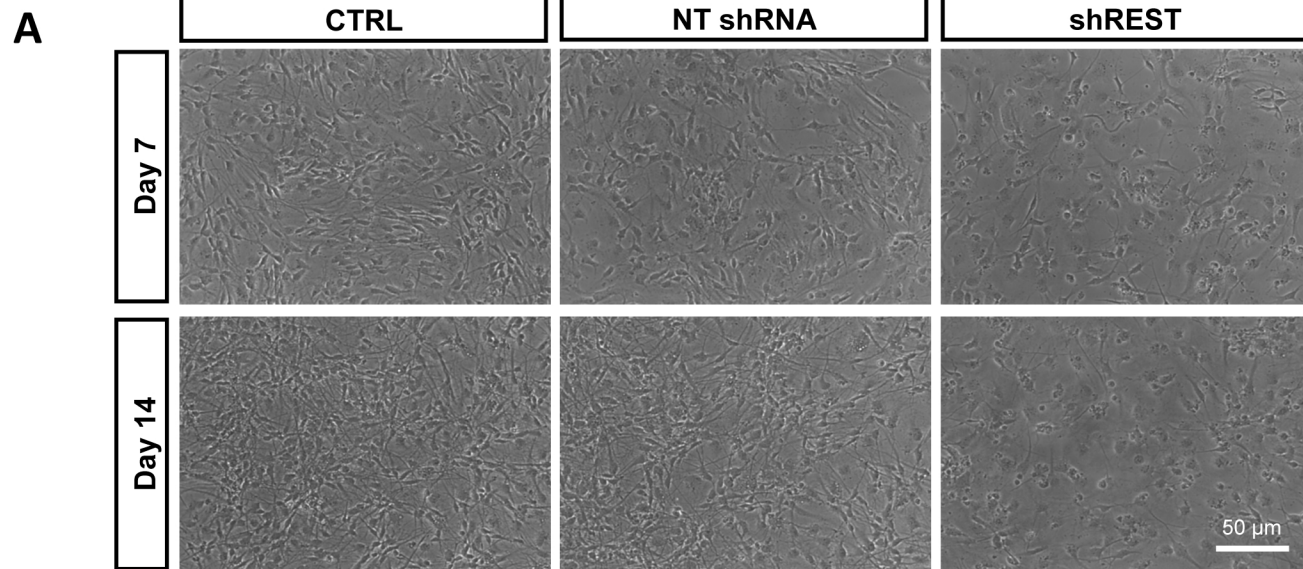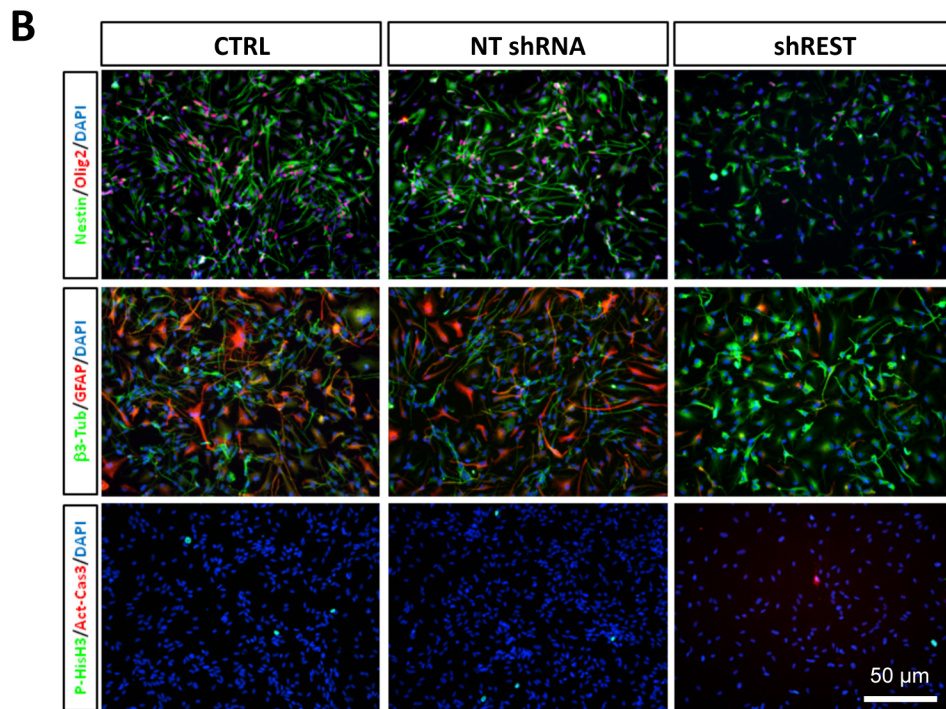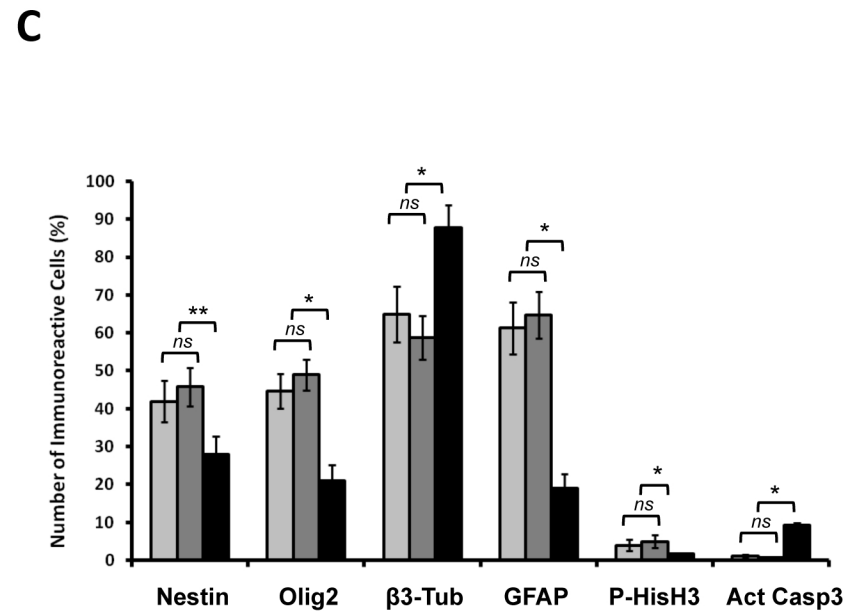

Supplement: Figure S8 — REST knockdown triggers neuronal differentiation and cell death programs in human tumorigenic-competent GBM cells. (A) Representative live image of GB7 cells exposed to differentiating conditions for 7 and 14 days after lentiviral REST shRNA knockdown (shREST) and relative controls (CTRL: untreated; NT shRNA: non targeting shRNA). REST knockdown produces drop of proliferation and dramatic morphological changes, with appearance of a pronounced proportion of flat and differentiated cells. (B) Immunofluorescent analyses of control(s) and shREST GB7 cells in differentiation contitions (14 days post lentiviral shRNA infection). The number of cells immunopositive for neural progenitor (Nestin and Olig2) and proliferation (P-HisH3) markers in shREST cultures is strongly lessened with respect to controls (CTRL and NT shRNA groups), with a parallel increase of neuronal differentiation (β3-tubulin) and apoptosis (Activated Caspase 3) markers. The number of GFAP expressing cells is reduced in shREST cultures with respect to the control cultures, indicating that REST derepression strongly favors the conversion toward the neuronal lineage at the expenses of the glial lineage. (C) Relative quantification of the numbers of immunoreactive cells in (B). CTRL: light gray bars; NT shRNA: dark gray bars; shREST: black bars. At least 700 cells per group were scored. Results shown are relative to three independent experiments. Data are means ± s.d. ns not significant, *P<0.001, **P<0.05. (PDF) [file pone.0038486.s008.pdf]

**A**

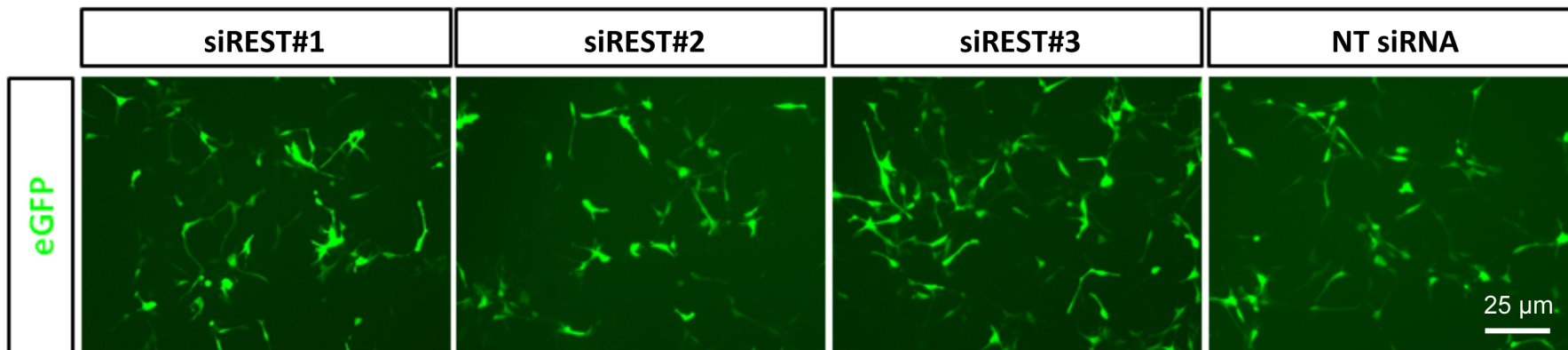

**B**

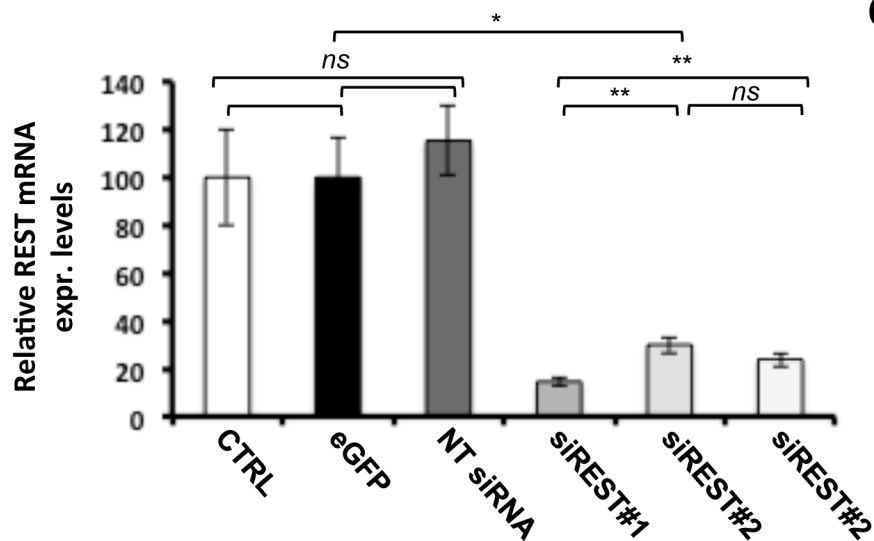

**C**

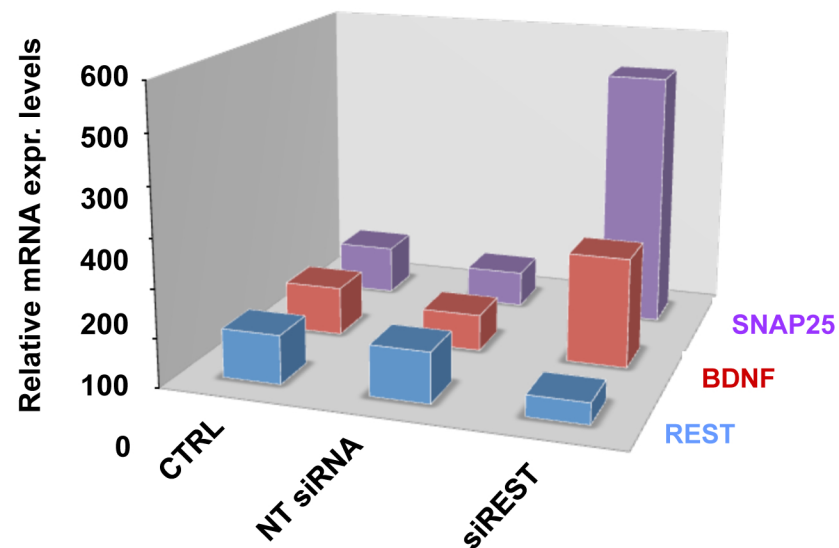

Supplement: Figure S9 — REST siRNA silencing efficiency in GB cells. (A) Live fluorescent images of GB7 cells 48 hours after nucleofection with three different anti REST siRNAs (siREST#1, siREST#2, siREST#3) each directed against a distinct region of REST transcript or with non targeting control siRNA (NT siRNA) and co-transfected with a GFP-carrying plasmid (eGFP) to assess nucleofection efficiency. The transfection efficiency and the fluorescence levels on siREST cultures are comparable to control cultures. Average nucleofection efficiency: 74.6% ±8.3. (B) REST siRNA targeting efficiently reduces REST transcript levels in GB cells. Real Time PCR analysis showing REST expression in siREST nucleofected GB7 cells and in control (CTRL, eGFP and NT siRNA) cultures. REST expression level was normalized to GAPDH. REST transcript level in CTRL group is considered as 100%. Best knockdown efficiency is achieved with siREST#1 (siREST#1: 17.3% ±3.5, P<0.001; siREST#2: 29.7% ±5.1, P<0.001; siREST#3: 24.2% ±4.4, P<0.001) which was selected for further experiments. No effect on REST levels after nucleofection in NT siRNA (116.4±15.6, P not significant) or GFP (98.3±17.1, P not significant) cultures. Data are relative of three independent experiments and are presented as mean ± s.d. (C) REST knockdown de-represses the REST-mediated silencing activity on target genes transcription in GB cells. Quantitative Real Time PCR analysis of transcripts levels of REST-controlled genes (BDNF and SNAP25) in siREST nucleofected (96 hours after transfection) and control GB7 cultures. RNA was purified from GB7 cells nucleofected with either anti-REST siRNA #1 (siREST#1) or non-targeting control siRNA (NT siRNA). RNA extracted from mock nucleofected cells was used as control (CTRL). GAPDH was used as housekeeping gene to normalize REST expression levels. The mRNA expression levels of CTRL group was set to 100 for each gene and data are shown as percentage of CTRL. REST targeting leads to a marked de-repression of REST-t [file pone.0038486.s009.pdf]

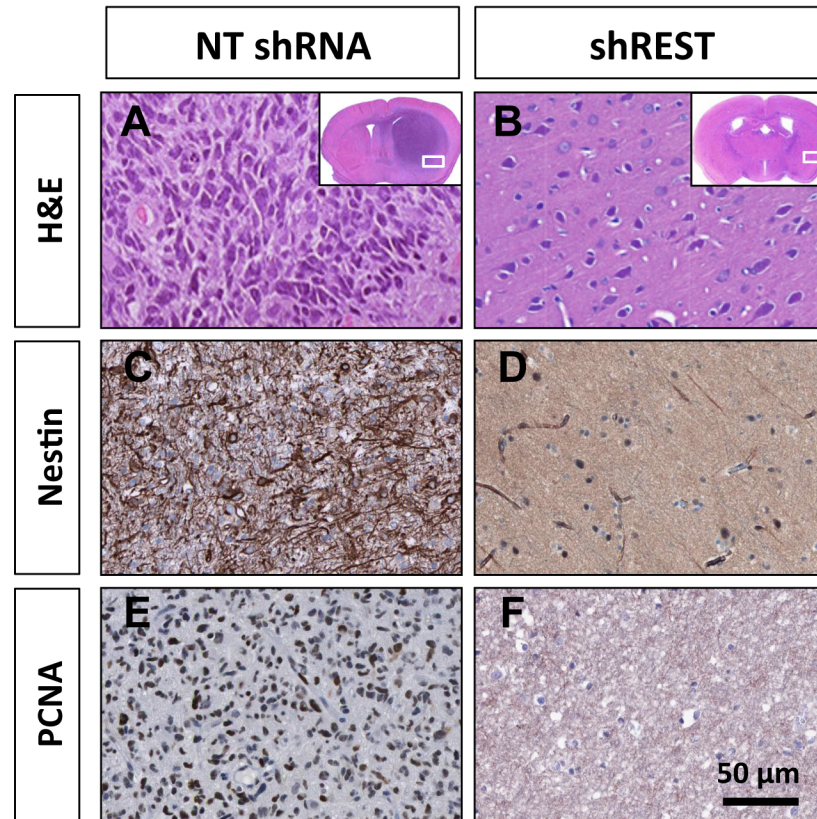

Supplement: Figure S10 — REST knockdown abolishes orthotopic xenograft tumor formation by human tumorigenic-competent GBM cells. GB7 cells transduced with NT shRNA or shREST lentiviral particles were injected into brains of SCID mice (150,000 cells per mouse; prior to transplantation, cultures were puromycin-selected for three days in order to eliminate non-infected cells). Four mice were injected for each group. Mice in the control group were sacrificed upon the development of neurologic signs. All the mice bearing shREST GB7 cells did not develop neurologic signs and were sacrificed after 180 days without evidence of tumor formation. (A and B) Representative images of coronal sections of grafted brains. H&E staining demonstrated the presence of brain tumors in mice injected with NT shRNA GB7 cells (A), while no tumors were observed in brains of mice injected with shREST GB7 cells (B). Insets in (A and B) show whole sections of the grafted brains. (C–F) Immunohistochemical staining of brain sections from NT shRNA mice (C and E) and shREST mice (D and F) with antibodies against nestin (C and D) and PCNA (E and F). (PDF) [file pone.0038486.s010.pdf]

**A**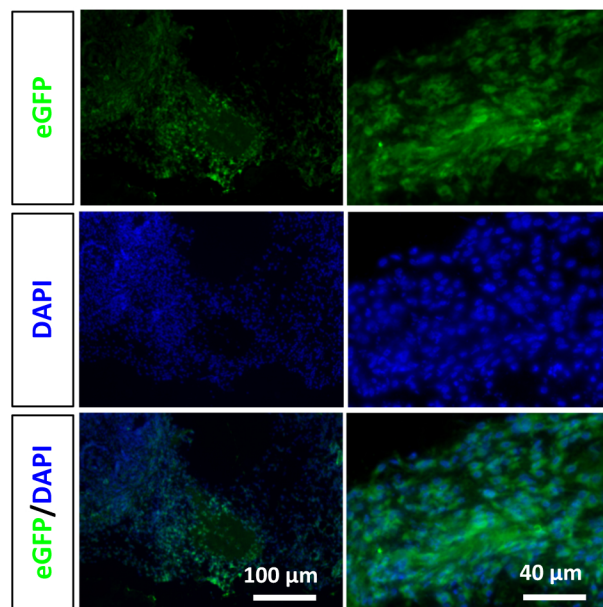**B**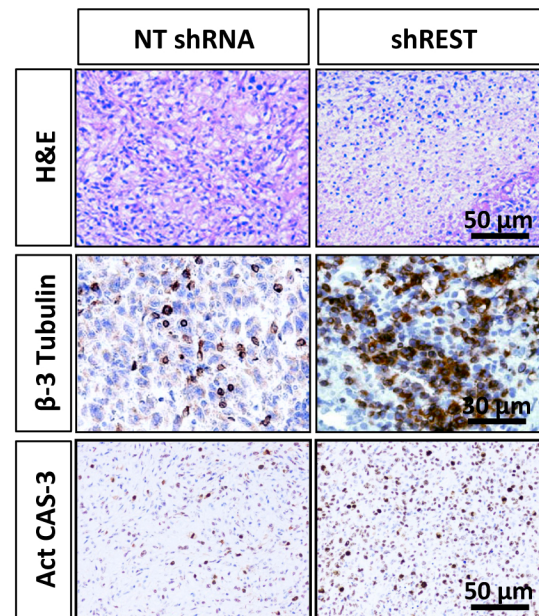

Supplement: Figure S11 — In vivo intra-tumoral injection of REST shRNA impairs growth of heterotopic established tumors. (A) In vivo lentiviral injection of control GFP-expressing lentiviral particles in established GB7 cells-derived xenograft tumors in SCID mice. GB7 cells were subcutaneously implanted into mice flanks and, once tumors were well established (42 days after grafting), GFP-expressing lentiviral particles were delivered to the tumor site through direct single injection in order to assess the infection efficiency. One-week post virus injection mice were sacrificed and tumor tissue sectioned. Representative fluorescent images of tumor section show the presence of eGFP signal indicative for the occurrence of a local infection of the tumor mass. (B) Lentiviral particles carrying either non-targeting shRNA or shRNA directed against REST were delivered to tumor site (42 days old GB7 cell-derived xenografts; n = 4 for each group) through direct injection (two injections with 7 days interval). Fifteen days after the last virus injection, mice were sacrificed and tumor tissue sectioned. H&E staining of tumor tissue sections enlightens the presence of areas with reduced cellular density in the shREST injected tumors, indicating focal infected areas where possibly cell death has occurred. The tissue from NT shRNA infected tumors shows a more uniform cellular density. Representative immunohistochemical staining of tumor tissue sections from NT shRNA and shREST groups with antibodies against β3-tubulin and Active Caspase-3 show indeed the presence of immunoreactive areas in shREST infected tumors. (PDF) [file pone.0038486.s011.pdf]
